# Patterns of adiposity, vascular phenotypes and cognitive function in the 1946 British Birth Cohort

**DOI:** 10.1186/s12916-018-1059-x

**Published:** 2018-05-28

**Authors:** Stefano Masi, Georgios Georgiopoulos, Tauseef Khan, William Johnson, Andrew Wong, Marietta Charakida, Peter Whincup, Alun D. Hughes, Marcus Richards, Rebecca Hardy, John Deanfield

**Affiliations:** 1 National Centre for Cardiovascular Prevention and Outcomes, Institute of Cardiovascular Science, University College London, 1 St Martin le Grande, London, EC1A 4NP UK; 2 Department of Clinical and Experimental Medicine, University of Pisa, Pisa, Italy; 3 First Department of Cardiology|, Hippokration Hospital, University of Athens, Athens, Greece; 4 School of Sport, Exercise and Health Sciences, Loughborough University, Loughborough, Leicestershire, UK; 5 MRC Unit for Lifelong Health and Ageing at UCL, Institute of Epidemiology and Health Care, UCL, London, UK; 6 Division of Population Health Sciences and Education, St George’s University of London, London, UK; 7 Cardiometabolic Phenotyping Group, Institute of Cardiovascular Science, UCL, London, UK

**Keywords:** Obesity, waist circumference, cognitive function, vascular phenotypes, lifetime risk

## Abstract

**Background:**

The relationship between long-term exposure to whole body or central obesity and cognitive function, as well as its potential determinants, remain controversial. In this study, we assessed (1) the potential impact of 30 years exposure to different patterns of whole body and central adiposity on cognitive function at 60–64 years, (2) whether trajectories of central adiposity can provide additional information on later cognitive function compared to trajectories of whole body adiposity, and (3) the influence of vascular phenotypes on these associations.

**Methods:**

The study included 1249 participants from the prospective cohort MRC National Survey of Health and Development. Body mass index (BMI), waist circumference (WC), and vascular (carotid intima-media thickness, carotid-femoral pulse wave velocity) and cognitive function (memory, processing speed, reaction time) data, at 60–64 years, were used to assess the associations between different patterns of adult WC or BMI (from 36 years of age) and late midlife cognitive performance, as well as the proportion of this association explained by cardiovascular phenotypes.

**Results:**

Longer exposure to elevated WC was related to lower memory performance (*p* < 0.001 for both) and longer choice reaction time (*p* = 0.003). A faster gain of WC between 36 and 43 years of age was associated with the largest change in reaction time and memory test (*P* < 0.05 for all). Similar associations were observed when patterns of WC were substituted with patterns of BMI, but when WC and BMI were included in the same model, only patterns of WC remained significantly associated with cognitive function. Participants who dropped one BMI category and maintained a lower BMI had similar memory performance to those of normal weight during the whole follow-up. Conversely, those who dropped and subsequently regained one BMI category had a memory function similar to those with 30 years exposure to elevated BMI. Adjustment for vascular phenotypes, levels of cardiovascular risk factors, physical activity, education, childhood cognition and socioeconomic position did not affect these associations.

**Conclusions:**

Longer exposure to elevated WC or BMI and faster WC or BMI gains between 36 and 43 years are related to lower cognitive function at 60–64 years. Patterns of WC in adulthood could provide additional information in predicting late midlife cognitive function than patterns of BMI. The acquisition of an adverse cardiovascular phenotype associated with adiposity is unlikely to account for these relationships.

**Electronic supplementary material:**

The online version of this article (10.1186/s12916-018-1059-x) contains supplementary material, which is available to authorized users.

## Background

The increasing prevalence of obesity represents a major public health concern, as it is associated with an increased risk of several chronic diseases, including cardiovascular disease (CVD). Several reports have suggested that exposure to whole body and abdominal obesity could influence cognitive function and risk of dementia, although results are conflicting and limited to older cohorts [1–7]. A recent study found that gene variants associated with greater body mass index (BMI) are also related to lower cognitive function [8], supporting the hypothesis that shared biological pathways could increase the risk of obesity and cognitive dysfunction. However, whether the association of obesity with cognitive outcomes is related to the cumulative burden of exposure or vulnerability to the effects of rapid changes of whole body or abdominal fat at specific stages of adulthood remains unclear.

Several factors could account for the association between amount and distribution of body fat and reduced cognitive function. A lifetime exposure to obesity is associated with the acquisition of an adverse cardiovascular phenotype. Carotid-to-femoral pulse wave velocity (PWV) and common carotid artery intima-media thickness (cIMT) are validated surrogate markers of arterial stiffness and atherosclerotic CVD [9, 10], and are known to be affected by exposure to whole body and abdominal obesity [11–14]. In turn, increased cIMT and PWV are associated with lower cognitive performance [15–20] and with a higher burden and rate of deposition of β-amyloid in the brain [21, 22]. Therefore, an altered vascular phenotype identified by greater cIMT or PWV could contribute to the association between adiposity and cognitive performance, but this has not been studied.

The MRC National Survey of Health and Development (NSHD, also known as the 1946 British Birth Cohort) is the oldest of the British Birth Cohort studies [23], and is unique in providing measures of BMI and waist circumference (WC) across the entire life course, together with a characterisation of vascular phenotypes, cardiovascular risk factors and cognitive function at 60–64 years. Using this population, we have previously explored the impact of BMI and its change over time on cognitive function at the age of 53 years [24]. We now extend this work to investigate whether central adiposity has an effect over and above the effect of general adiposity on cognitive function at 60–64 years of age. We also assess whether rapid changes of BMI or WC over different periods of adult life can have a specific influence on cognitive function at 60–64 years of age. Finally, we explored what proportion, if any, of the association between adult patterns of adiposity and cognitive function could be explained by acquisition of an adverse cardiovascular phenotype.

## Methods

### Population

The MRC NSHD is a nationally representative sample of 5362 singleton births to married parents in England, Scotland and Wales, stratified by social class, during 1 week in March 1946 [25, 26]. The cohort has been followed-up 23 times from birth to age 69 years. The present study is based on the 1249 (74%) of 1690 participants who had adiposity measures, vascular phenotype and cognitive data at 60–64 years with a BMI > 18.5 kg/m^2^. Further details on the sample invited at the 60–64 years assessment are provided in Additional file 1.

### Cognitive assessment

Cognitive function was assessed at age 60–64 years using a validated verbal memory test, a letter search speed test and two reaction time tests (simple reaction time, choice reaction time) [24]. Details of each cognitive test are provided in Additional file 1.

### Adiposity measures

Weight, height and WC were measured during adulthood at ages 36, 43, 53 and 60–64 years. BMI was calculated as weight (kg) divided by squared height (m^2^), and was used to define adiposity status according to World Health Organization criteria (BMI 18.5–25 kg/m^2^ normal weight, 25–29 kg/m^2^ overweight, and ≥ 30 kg/m^2^ obese) at each age. Similarly, we identified three classes of cardiometabolic risk related to WC, namely (1) low risk = WC ≤ 94 cm for males and ≤ 80 cm for females; (2) increased risk = WC > 94 cm and ≤ 102 cm for males and > 80 cm and ≤ 88 cm for females; (3) substantially increased risk = WC > 102 cm for males and > 88 cm for females [27]. Participants in the classes defined as increased and substantially increased risk were combined into an elevated WC group.

### Vascular phenotypes

At age 60–64 years, PWV and cIMT were measured using validated devices and following standard protocols [28, 29], as reported in Additional file 1.

### Covariates

Covariates were either selected a priori or were those variables that were associated with cognitive measures in univariable models. These included level of education, childhood cognition, socioeconomic position, heart rate, systolic blood pressure, smoking, diabetes and its duration, total cholesterol, and levels of physical activity. Methods used for their assessment are reported in Additional file 1.

### Statistical analysis

Mean (standard deviation), or median (IQR) for skewed variables, were used to describe continuous variables and percentages for binary variables. We tested for effect modification by sex of obesity indices on cognitive outcomes by introducing relevant interaction terms (BMI*sex) in multivariable regression models. Level of statistical significance for interaction terms was set at 0.1 and, when a significant interaction was found, results were stratified by sex. We fitted a series of linear multivariable regression models to establish the associations between (1) BMI at 60–64 years, (2) patterns of overweight/obesity, and (3) conditional change in BMI from 36 to 43, 43 to 53, and 53 to 60–64 with each cognitive outcome and with each vascular phenotype (PWV, cIMT). In each model, inverse probability weighting was implemented to account for dropout due to death. The models were sequentially adjusted for covariates: MODEL 1 adjusted for sex, education and childhood cognition; MODEL 2 = MODEL 1 + socioeconomic position at 53 years, systolic blood pressure and heart rate at age 60–64; MODEL 3 (fully adjusted) = MODEL 2 + total cholesterol, smoking, diabetes and levels of physical activity at 60–64 years. In analyses exploring the association between patterns or conditional changes of BMI with cognitive outcomes (2 and 3), MODEL 3 was further adjusted for duration of diabetes. We also tested for effect modification by socioeconomic position and education levels of the association between BMI changes and cognitive outcomes. All multivariable regression models of cognitive outcomes on cross-sectional measures or longitudinal patterns of BMI were further adjusted for PWV and, separately, cIMT to explore what proportion of these associations could be explained by cardiovascular phenotype. Each analysis was repeated using WC rather than BMI as the exposure. To assess whether central adiposity had an effect over and above the effect of general adiposity, each cross-sectional and longitudinal analysis was repeated including BMI and WC in the same models. Further details on the statistical methods are reported in Additional file 1. Statistical analyses were performed using Stata 13.1.

## Results

Table 1 reports the characteristics of the total sample used in these analyses (*n* = 1249) as well as the differences between groups of normal weight, overweight and obese at 60–64 years old. We have previously reported that the sample attending the clinical research facility showed some differences in characteristics compared with those not attending [30].

**Table 1:** Participant characteristics by BMI category at the age of 60–64 years

	N	Entire sample	Normal (30.5%)	Overweight (42.5%)	Obese (27.0%)	*p* value trend
Male sex (%)	1249	46.3	37.0	54.7	43.8	*0.046*
Height (m)	1249	1.68 (0.1)	1.68 (0.1)	1.69 (0.1)	1.67 (0.1)	0.44
Weight (kg)	1249	78.4 (14.9)	65.3 (8.2)	78.1 (9.2)	94.3 (12.5)	**<** *0.001*
BMI (kg/m^2^)	1249	27.6 (4.6)	23.0 (1.5)	27.2 (1.4)	33.6 (3.6)	*< 0.001*
Waist circumference (cm)	1249	96.6 (12.6)	84.6 (8.2)	96.0 (7.6)	109.2 (9.8)	*< 0.001*
Hip circumference (cm)	1249	105.8 (9.5)	97.7 (4.9)	104.5 (5.1)	115.7 (9.0)	*< 0.001*
Waist-to-hip ratio	1249	0.91 (0.1)	0.87 (0.1)	0.92 (0.1)	0.95 (0.1)	*< 0.001*
Heart rate (bpm)	1248	68.4 (11.3)	67.2 (11.4)	68.3 (10.6)	69.8 (12.1)	*0.009*
SBP (mmHg)	1248	134.8 (17.9)	129.3 (16.7)	136.6 (18.1)	138 (17.9)	*< 0.001*
DBP (mmHg)	1248	77.2 (9.7)	74.5 (9.4)	78.2 (9.4)	78.7 (10.1)	*< 0.001*
Education (% above A-level)	909	61.3	61.9	65	54.6	0.14
Smoking (% current smokers)	1171	34.9	37.7	33.7	34.3	0.34
Total cholesterol (mmol/L)	1186	5.69 (1.2)	5.91 (1.1)	5.7 (1.2)	5.4 (1.3)	*< 0.001*
LDL cholesterol (mmol/L)	1156	3.52 (1.0)	3.66 (0.9)	3.55 (1.0)	3.3 (1.1)	*0.001*
HDL cholesterol (mmol/L)	1186	1.62 (0.41)	1.81 (0.4)	1.59 (0.4)	1.43 (0.3)	*< 0.001*
Triglycerides^a^ (mmol/L)	1162	1.1 (0.8–1.5)	0.8 (0.6–1.1)	1.1 (0.8–1.5)	1.3 (1.0–1.9)	*< 0.001*
HbA1c^a^ (%)	1157	5.8 (5.5–6)	5.7 (5.5–5.9)	5.7 (5.5–6.0)	5.9 (5.6–6.2)	*< 0.001*
Adiponectin^a^ (μg/mL)	1183	12.9 (7.6–19.5)	16.6 (10.9–25.1)	12.1 (7.0–17.8)	10.2 (6.3–16.4)	*< 0.001*
Leptin^a^ (ng/mL)	1183	12.1 (6.6–23.6)	7.8 (4.1–13.7)	11.6 (6.4–19.1)	26.1 (14.1–43.2)	*< 0.001*
Levels of physical activity (in the last 4 weeks)						
• Not physical active • Moderate • Intense	1223	703 (58.15) 189 (15.63) 317 (26.22)	205 (54.81) 61 (16.31) 108 (28.88)	297 (58.01) 80 (15.63) 135 (26.37)	201 (62.23) 48 (14.86) 74 (22.91)	0.375
PWV (m/s)	1249	8.2 (1.51)	7.95 (1.58)	8.27 (1.5)	8.37 (1.42)	*< 0.001*
cIMT (mm)	900	0.69 (0.12)	0.67 (0.12)	0.69 (0.13)	0.71 (0.11)	*<0.001*
VMT (number of words)	1227	24.7 (6.1)	26.0 (6.0)	24.5 (6.2)	23.6 (5.7)	*< 0.001*
LSST^a^ (targets)	1249	282 (231–307)	287 (231–329)	239 (231–329)	263 (174–288)	*0.009*
S-RT (s)	1234	281 (63.5)	279 (64.5)	278 (60.2)	289 (67.2)	*0.038*
C-RT (s)	1230	612 (76.5)	607 (77.4)	608 (72.9)	622 (80.6)	*0.012*

Values are presented as mean ± standard deviation, N (%) or ^a^median (IQR). Comparisons between normal weight, overweight and obese groups were performed by test for trend using linear regression

Numbers in italic indicate statistical significance

*SBP* systolic blood pressure, *DBP* diastolic blood pressure, *cIMT* common carotid artery intima-media thickness, *PWV* pulse wave velocity, *VMT* verbal memory test, *LSST* letter search speed test, *C-RT* choice reaction time test, *S-RT* simple reaction time test

### Cross-sectional analysis

#### Association of BMI and WC with cognitive function at 60–64 years

We did not find any significant effect modification of obesity indices on cognitive outcomes by sex, apart from the letter search speed test. In models adjusted for sex, education and childhood cognition, BMI was positively associated with cIMT (regression coefficient (β) = 0.003 mm per kg/m^2^; 95% confidence interval (CI) 0.001 to 0.005; *p* = 0.01) and PWV (β = 0.040 m/sec per kg/m^2^; 95% CI 0.012 to 0.068; *p* = 0.005) (Table 2). The association of BMI with cIMT remained significant in the fully adjusted model, while that with PWV was attenuated in MODEL 2 and further reduced in the final model (Table 2). A higher BMI was associated with a lower performance on the verbal memory test (β = −0.195 number of words per kg/m^2^; 95% CI –0.274 to −0.116; *p* < 0.001) and the letter search speed test (β = −0.005 number of targets hit per kg/m^2^; 95% CI –0.010 to −0.001; *p* = 0.018). When stratified by sex, the association between higher BMI and lower letter search speed test performance was stronger in females than in males (Additional file 1: Table S1). These associations remained significant in the fully adjusted models. No associations were observed between BMI and choice reaction time or simple reaction time (Table 2). Additional file 1: Table S2 reports the associations between WC and the vascular and cognitive measures at 60–64 years. WC was associated with vascular phenotypes, verbal memory test performance and, differently from BMI, with choice reaction time, but not with performance in the letter search speed test. When BMI and WC were included in the same model, the associations of both with performance in the verbal memory test and letter search speed test were strongly attenuated, suggesting that the two measures of adiposity provided similar information. In turn, WC remained significantly associated with choice reaction time after adjustment for BMI (β = 6.73; 95% CI 1.32 to 12.1; *p* = 0.015).

**Table 2:** Cross-sectional associations of BMI with carotid intima-media thickness and pulse wave velocity with verbal memory, letter search speed, choice and simple reaction test at age 60–64 years

	MODEL 1		MODEL 2		MODEL 3	
	β (95% CI)	*p*	β (95% CI)	*p*	β (95% CI)	*p*
VASCULAR MEASURES						
1. cIMT (mm)	*0.003 (0.0003 to 0.005)*	*0.025*	0.002 (−0.0003 to 0.005)	0.085	*0.003 (0.0004 to 0.006)*	*0.024*
2. PWV (m/s)	*0.040 (0.012 to 0.068)*	*0.005*	0.018 (−0.009 to 0.045)	0. 192	0.014 (−0.015 to 0.043)	0.355
COGNITIVE MEASURES						
1. VMT (n. of words)	*–0.195 (−0.274 to −0.116)*	*< 0.001*	*−0.178 (−0.255 to −0.098)*	*< 0.001*	*−0.162 (−0.248 to −0.076)*	< *0.001*
2. LSST^a^ (targets)	*–0.005 (−0.010 to −0.001)*	*0.018*	*−0.005 (−0.010 to −0.001)*	*0.027*	−0.004 (−0.009 to 0.001)	0.117
3. RT (s)						
a. Simple	0.485 (−0.373 to 1.344)	0.267	0.552 (−0.324 to 1.428)	0.217	0.689 (−0.300 to 1.679)	0.172
b. Choice	0.910 (−0.222 to 2.042)	0.115	0.983 (−0.157 to 2.123)	0.091	1.014 (−0.292 to 2.320)	0.128

Linear regression models were used to assess associations between variables

MODEL 1 adjusted for sex, education, childhood cognition; MODEL 2 = MODEL 1 + adjustments for socioeconomic position at age 53, systolic blood pressure and heart rate at age 60–64; MODEL 3 (fully adjusted) = MODEL 2 + adjustments for total cholesterol, smoking, diabetes and levels of physical activity. In each model, inverse probability weighting was implemented to account for the probability of survival until the end of follow-up

Numbers in italics indicate statistical significance

*cIMT* common carotid artery intima-media thickness, *PWV* pulse wave velocity, *VMT* verbal memory test, *LSST* letter search speed test, *RT* reaction time test

^a^Indicates log transformed dependent variables

#### Associations of cardiovascular risk factors/phenotypes with cognitive function at 60–64 years

Associations between cardiovascular risk factors and each cognitive outcome are reported in Additional file 1: Table S3. Higher PWV was associated with a lower performance in the verbal memory test (β = −0.355 number of words per m/s; 95% CI –0.616 to −0.095; *p* = 0.008), with the strength of this association attenuated in MODEL 3, while no association was found between cIMT and verbal memory test performance (Table 3). The letter search speed and both reaction time tests were not associated with any vascular phenotype. Substitution of pulse pressure for systolic blood pressure in MODELS 2 and 3 did not affect the association between PWV and verbal memory test. As PWV was associated with both BMI and memory function, we tested whether the association between BMI and verbal memory test performance was attenuated by PWV, suggesting that PWV may be a mediator of the association between adiposity and memory performance. However, adjustment for PWV only slightly attenuated the association between BMI and verbal memory test in the fully adjusted model (unadjusted for PWV: β = −0.172 number of words per kg/m^2^; 95% CI –0.258 to −0.086 vs. adjusted for PWV β = −0.170 number of words per kg/m^2^; 95% CI –0.256 to −0.084; proportional difference in β = 1.3%).

**Table 3:** Cross-sectional associations of carotid intima-media thickness and pulse wave velocity with verbal memory test, letter search speed and reaction time at 60–64 years

		VMT (n. of words)		LSST ^a^ (targets)		S-RT (s)		C-RT (s)	
		β (95% CI)	*p*	β (95% CI)	*p*	β (95% CI)	*p*	β (95% CI)	*p*
PWV	Model 1	*−0.355 (−0.616 to −0.095)*	*0.008*	0.004 (−0.018 to 0.026)	0.701	−0.958 (−3.676 to 1.760)	0.489	−1.871 (−5.485 to 1.741)	0.309
	Model 2	*−0.297 (−0.578 to −0.015)*	*0.039*	0.006 (−0.020 to 0.032)	0.629	−0.344 (−3.402 to 2.714)	0.825	−1.357 (−5.270 to 2.557)	0.496
	Model 3	−0.166 (0.461 to 0.129)	0.270	0.007 (−0.022 to 0.036)	0.634	−0.029 (−3.417 to 3.359)	0.987	−1.148 (−5.710 to 2.749)	0.492
cIMT	Model 1	0.220 (−3.470 to 3.912)	0.906	−0.036 (−0.218 to 0.145)	0.695	6.819 (−27.267 to 40.906)	0.694	16.606 (−32.294 to 65.507)	0.505
	Model 2	0.487 (−3.162 to 4.136)	0.793	−0.049 (−0.235 to 0.138)	0.607	10.673 (−24.072 to 45.418)	0.546	21.282 (−27.069 to 69.635)	0.388
	Model 3	1.287 (−2.340 to 4.913)	0.486	−0.015 (−0.222 to 0.192)	0.888	12.470 (−24.800 to 49.740)	0.511	25.184 (−25.895 to 76.264)	0.333

Linear regression models were used to assess associations between variables

*cIMT* common carotid artery intima-media thickness, *PWV* pulse wave velocity, *VMT* verbal memory test, *LSST* letter search speed test, *C-RT* choice reaction time test, *S-RT* simple reaction time test

MODEL 1 adjusted for sex, education and childhood cognition; MODEL 2 = MODEL 1 + adjustments for socioeconomic position at age 53, systolic blood pressure and heart rate at age 60–64; MODEL 3 (fully adjusted) = MODEL 2 + adjustments for total cholesterol, smoking, diabetes and levels of physical activity. In each model, inverse probability weighting was implemented to account for the probability of survival until the end of follow-up

^a^Indicates log transformed dependent variables

Numbers in italics indicate statistical significance

### Longitudinal analysis

#### Association of patterns of cumulative exposure to overweight or obesity and elevated WC in adulthood with cognitive function

**Fig. 1:**
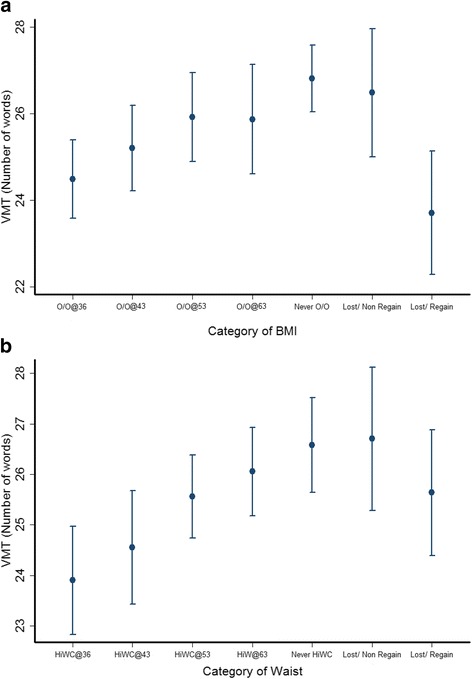
**a** Patterns of overweight/obesity change and performance in the verbal memory test (VMT) at age 60–64 years. Data points represent mean number of words recalled and vertical bars indicate 95% CI for each group. O/O@36, O/O@43, O/O@53 and O/O@60–64 = Overweight/obesity since 36, 43, 53 and 60–64 years old, respectively; Never O/O = never overweight/obese; Lost/Non-regain = dropped and did not regain one category of BMI; Lost/Regain = dropped and regained one category of BMI. VMT = Verbal memory test. Results are adjusted for sex, childhood cognition and education, and inverse probability weighting was implemented to account for dropout due to death. Test for trend for O/O@36 to O/O@60–64: *p* < 0.0001. Pairwise comparison of Never O/O versus Lost/Non-regain (*p* = 0.9) and Lost/Regained (*p* = 0.002). Pairwise comparison of O/O@36 versus Lost/Non-regain (*p* = 0.007) and Lost/Regain (*p* = 0.9). Adjustment for cardiovascular risk factors, PWV and cIMT did not affect these differences. **b** Patterns of waist circumference (WC) change and performance in the verbal memory test (VMT) at age 60–64 years. Data points represent mean number of words recalled and vertical bars indicate 95% CI for each group. HiWC@36, HiWC@43, HiWC@53 and HiWC@60–64 = elevated WC since 36, 43, 53 and 60–64 years old, respectively; Never HiWC = WC always normal; Lost/Non-regain = dropped and did not regain one category of WC; Lost/Regain = dropped and regained one category of WC. Results are adjusted for sex, childhood cognition and education, and inverse probability weighting was implemented to account for the probability of survival until the end of follow-up. Test for trend for HiWC@36 to HiWC@60–64: *p* < 0.001. Adjustment for cardiovascular risk factors, PWV and cIMT did not affect these differences

From age 36 to 60–64 years, the prevalence of overweight/obesity increased from 29% to 69%. Of 1021 participants with vascular phenotypes at 60–64 years and complete BMI records at all ages, 141 (14%) had a reduction in BMI category during 27 years of follow-up; 78 (8%) subsequently regained weight, leaving 63 (6%) with stable weight reduction. Earlier onset of overweight/obesity was associated with a worse cardiometabolic profile and higher PWV and cIMT (Additional file 1: Table S4). There was a graded relationship between increasing length of time being overweight/obese and decreasing verbal memory test performance (β = −0.752 words per category of increasing length of time overweight/obese; 95% CI –1.157 to −0.346; *p* for trend < 0.001). Individuals who were classified as overweight/obese at age 36 years recalled 2.3 (95% CI −3.5 to −1.1) fewer words compared to those who were always normal (Fig. 1a). Participants who were able to drop one BMI category and maintain a lower BMI had a similar verbal memory test performance to those who had never been overweight or obese. Conversely, those who dropped one BMI category but who subsequently moved up a category had a verbal memory test score similar to those with onset of overweight/obesity at 36 years old. There was no association between patterns of overweight/obesity and letter search, simple and choice reaction time tests (Additional file 1: Figures S1 and S2).

Full adjustment for cardiovascular risk factors (MODEL 3) only slightly reduced the association between increased duration of overweight/obesity exposure and verbal memory test (β = −0.666 words per category of increasing length of time being overweight/obese; 95% CI –1.119 to −0.213; *p* for trend = 0.004). Further adjustment of this association by PWV resulted in a 0.9% change in the β coefficient (β = −0.672 words per category of increasing length of time being overweight/obese; 95% CI –1.125 to −0.220; *p* for trend = 0.0037), and a 6.9% change of the β coefficient was observed when the same association was adjusted for cIMT (β = −0.620 words per category of increasing length of time being overweight/obese; 95% CI –1.176 to −0.063; *p* for trend = 0.029). When added to the model, the interaction between medication use and overweight/obese groups was not significant, and exclusion of participants with previous CVD history did not substantially affect results.

Analyses were repeated using patterns of WC in place of BMI. Similar to findings for BMI, longer exposure to elevated WC was associated with a worse cardiometabolic profile and higher PWV and cIMT (Additional file 1: Table S5). Greater length of time with elevated WC was associated with decreases in verbal memory test score, so that those with elevated WC from age 36 years had the lowest mean scores (β = −0.980 words per category of increasing length of exposure to elevated WC; 95% CI –1.424 to −0.536; *p* for trend < 0.001) (Fig. 1b); this association remained in the fully adjusted model (β = −1.051; 95% CIs −1.532 to −0.571, *p* for trend < 0.001). Individuals who were able to drop and not regain one category of WC had a similar memory performance as the group who always had a normal WC. Further adjustment of MODEL 3 for PWV resulted in a 0.8% decrease in the regression coefficient (β = −1.043 words per category of increasing length of exposure to elevated WC; 95% CIs −1.529 to −0.556; *p* for trend < 0.001) and a 13.8% decrease was observed when the same association was adjusted for cIMT (β = −0.906 words per category of increasing length of exposure to elevated WC; 95% CI –1.508 to −0.304; *p* for trend = 0.003). No clear trends were observed for the associations between categories of WC and letter search speed or simple reaction time tests (Additional file 1: Figures S3 and S4A). For choice reaction time, participants with high WC at 53 years and before had slower times than those with raised WC at 60–64 and those never having had a raised WC (β = 14.115 words per category of increasing length of exposure to elevated WC; 95% CI 6.453 to 21.776; *p* for trend = 0.003) (Additional file 1: Figure S4B).

When both BMI and WC were included in the same fully adjusted model, the linear trend across categories of exposure to elevated WC for verbal memory test (β = −0.871 per change in WC category; 95% CI −1.720 to −0.024, *p* for trend = 0.044) and choice reaction time test (β = 14.087 per change in WC category; 95% CI 3.300 to 24.873; *p* for trend = 0.011) remained significant, while the association between categories of BMI and verbal memory test score was considerably reduced and no longer significant (β = −0.257 per change in BMI category; 95% CI –0.979 to 0.465, *p* = 0.485).

#### Association of BMI and WC gains in adulthood with cognitive function

A faster increase in BMI between 53 to 60–64 years was related to lower verbal memory test score at age 60–64 (Additional file 1: Figure S5A), but this association was attenuated in MODELS 2 and 3 (Table 4). A greater BMI increase between 36 and 43 years was related to lower log (letter search speed) at age 60–64 (Additional file 1: Figure S5B). This association remained highly significant in the fully adjusted model (Table 4), and was stronger in females than in males (Additional file 1: Table S8 and Figure S6S). Evidence of effect modification by social class was observed for the association between BMI change from 36 to 43 years and choice reaction time, such that this association was stronger in those from more advantaged social classes (Additional file 1: Table S9).

**Table 4:** Relationship between cognitive tests at age 60–64 years and residual changes in BMI between three time periods: BMI_36–43_ = 36–43 years, BMI_43–53_ = 43–53 years, and BMI_53–60 to 64_ = 53–60 to 64 years

	MODEL 1		MODEL 2		MODEL 3	
	β (95% CI)	*p*	β (95% CI)	*p*	β (95% CI)	*p*
VMT						
BMI_36–43_	−0.216 (−0.629 to 0.198)	0.306	−0.161 (−0.553 to 0.231)	0.421	−0.199 (−0.632 to 0.234)	0.367
BMI_43–53_	0.065 (−0.390 to 0.520)	0.780	0.068 (−0.378 to 0.514)	0.763	0.098 (−0.393 to 0.590)	0.694
BMI_53–60 to 64_	*−0.524 (−0.923 to −0.124)*	*0.010*	*−0.515 (−0.915 to −0.115)*	*0.012*	−0.380 (−0.839 to 0.078)	0.104
LSST^a^						
BMI_36–43_	*−0.023 (−0.044 to −0.002)*	*0.029*	*−0.022 (−0.044 to −0.002)*	*0.035*	*−0.024 (−0.047 to −0.002)*	*0.034*
BMI_43–53_	−0.012 (−0.033 to 0.010)	0.280	−0.011 (−0.033 to 0.010)	0.305	−0.008 (−0.031 to 0.014)	0.462
BMI_53–60 to 64_	0.005 (−0.019 to 0.030)	0.680	0.005 (−0.020 to 0.029)	0.704	0.013 (−0.016 to 0.043)	0.367
S-RT						
BMI_36–43_	2.116 (−1.973 to 6.206)	0.310	2.205 (−1.903 to 6.312)	0.290	3.701 (−0.836 to 8.238)	0.110
BMI_43–53_	1.638 (−2.437 to 5.714)	0.430	1.775 (−2.377 to 5.928)	0.402	2.387 (−2.079 to 6.853)	0.294
BMI_53–60 to 64_	0.698 (−3.119 to 4.514)	0.720	1.142 (−2.672 to 4.955)	0.557	1.859 (−2.514 to 6.233)	0.404
C-RT						
BMI_36–43_	−1.610 (−7.038 to 3.818)	0.561	−1.433 (−6.968 to 4.101)	0.611	−0.798 (−6.749 to 5.152)	0.792
BMI_43–53_	2.581 (−3.305 to 8.468)	0.389	3.145 (−2.879 to 9.170)	0.306	3.021 (−3.570 to 9.612)	0.368
BMI_53–60 to 64_	2.146 (−3.157 to 7.449)	0.427	2.716 (−2.537 to 7.969)	0.310	4.230 (−1.835 to 10.295)	0.171

Linear regression models were used to assess associations between variables. Significant associations (*p* < 0.05) are highlighted in bold

^a^Indicates log transformed dependent variables. MODEL 1 adjusted for sex, education and childhood cognition; MODEL 2 = MODEL 1 + adjustments for socioeconomic position at age 53, systolic blood pressure and heart rate at age 60–64; MODEL 3 (fully adjusted) = MODEL 2 + adjustments for total cholesterol, smoking, diabetes, diabetes duration and levels of physical activity. In each model, inverse probability weighting was implemented to account for dropout due to death

*VMT* verbal memory test, *LSST* letter search speed test, *S-RT* simple reaction time test, *C-RT* choice reaction time

**Fig. 2:**
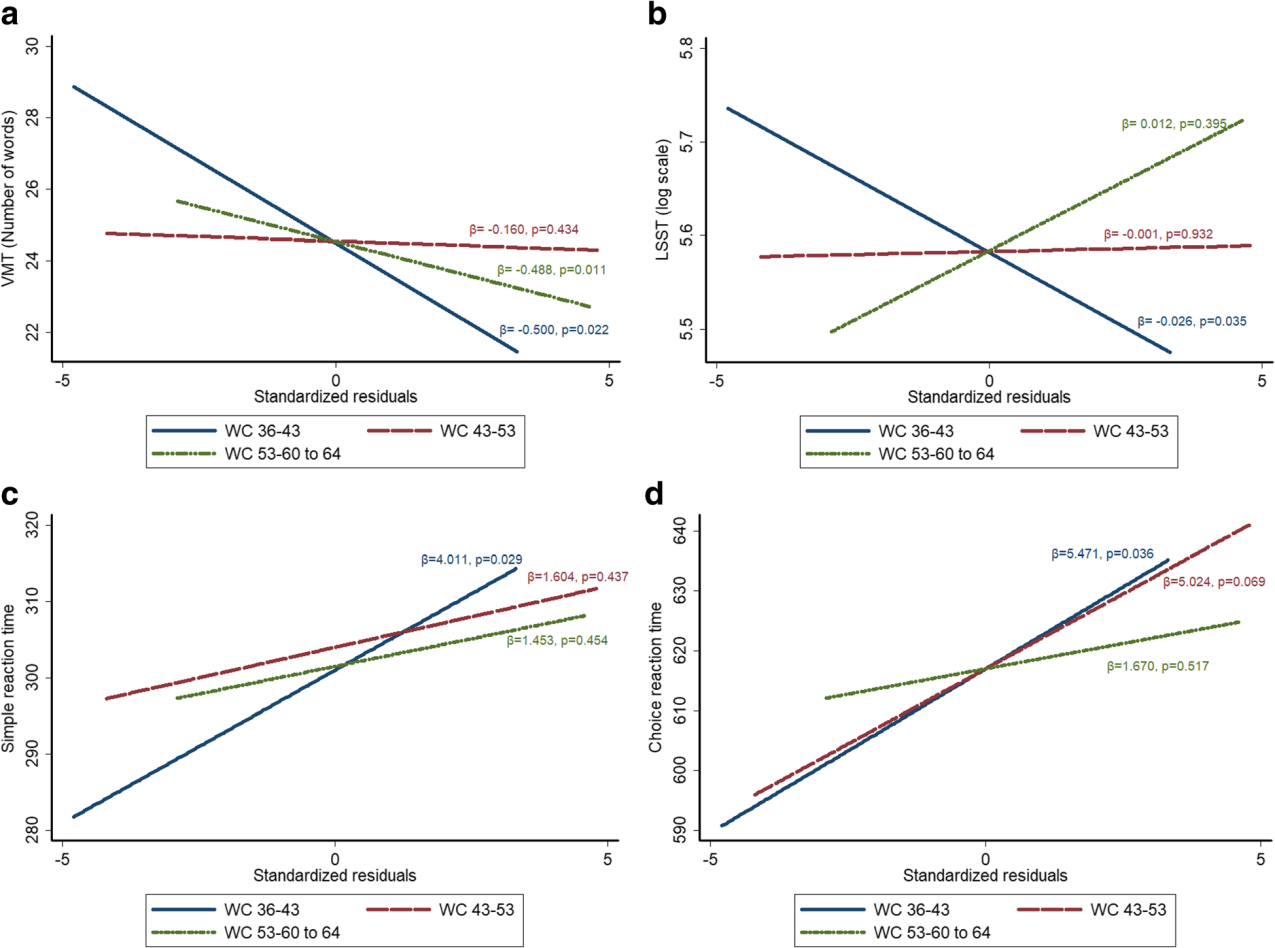
Association of rate of change in waist circumference (WC) in three time periods (36–43 years, 43–53 years and 53–60 to 64 years) according to MODEL 1 with (**a**) verbal memory test (VMT), (**b**) letter search speed test (LSST), (**c**) simple reaction time test and (**d**) choice reaction time (C-RT) at age 60–64 years. β represents the slope of the linear regression and indicates the difference in units of the cognitive outcome for 1 standard deviation increase in WC in each interval

A greater increase in WC between 36 and 43 years was associated with poorer performance in all cognitive tests at 60–64 years (Fig. 2a–d). The progressive adjustments from MODEL 1 to MODEL 3 minimally attenuated the strength of these associations (Table 5). As for BMI, a faster increase in WC between 53 to 60–64 years was related to lower verbal memory test score at age 60–64, but this association was attenuated in MODEL 3 (Table 5). There was no evidence of effect modification by socioeconomic position or education level of the association between WC increase and cognitive outcomes.

**Table 5:** Relationship between cognitive tests at age 60–64 years and residual changes in waist circumference between three time periods: Waist_36–43_ = 36–43 years, Waist_43–53_ = 43–53 years, and Waist_53–60 to 64_ = 53–60 to 64 years

	MODEL 1		MODEL 2		MODEL 3	
	β (95% CI)	*P*	β (95% CI)	*P*	β (95% CI)	*P*
VMT						
WC_36–43_	*−0.500 (−0.930 to −0.071)*	*0.022*	*−0.447 (−0.875, −0.019)*	*0.041*	*−0.501 (−0.972 to −0.030)*	*0.037*
WC_43–53_	−0.160 (−0.562 to 0.241)	0.434	−0.127 (−0.525 to 0.270)	0.530	−0.090 (−0.511 to 0.330)	0.672
WC_53–60 to 64_	*−0.488 (−0.863 to −0.113)*	*0.011*	*−0.444 (−0.822 to −0.067)*	*0.021*	−0.358 (−0.766 to 0.049)	0.085
LSST^a^						
WC_36–43_	*−0.026 (−0.051 to −0.002)*	*0.035*	*−0.025 (−0.050, −0.001)*	*0.045*	−0.027 (−0.055 to 0.001)	0.058
WC_43–53_	−0.001 (−0.026 to 0.024)	0.932	0.0003 (−0.025 to 0.025)	0.983	0.001 (−0.026 to 0.029)	0.916
WC_53–60 to 64_	0.012 (−0.016 to 0.040)	0.395	0.013 (−0.015 to 0.041)	0.372	0.018 (−0.014 to 0.051)	0.273
S-RT						
WC_36–43_	*4.011 (0.420 to 7.602)*	*0.029*	*4.140 (0.473 to 7.806)*	*0.027*	*4.435 (0.386 to 8.486)*	*0.032*
WC_43–53_	1.604 (−2.445 to 5.654)	0.437	1.718 (−2.408 to 5.844)	0.414	2.466 (−2.002 to 6.936)	0.279
WC_53–60 to 64_	1.453 (−2.357 to 5.263)	0.454	1.753 (−2.125 to 5.631)	0.375	2.196 (−2.120 to 6.512)	0.318
C-RT						
WC_36–43_	*5.471 (.356 to 10.588)*	*0.036*	*0.787 (0.599 to 10.976)*	*0.029*	*60.086 (0.389 to 11.783)*	*0.036*
WC_43–53_	5.024 (−0.390 to 10.439)	0.069	*5.530 (0.095 to 10.965)*	*0.046*	50.251 (−0.752 to 11.254)	0.086
WC_53–60 to 64_	1.670 (−3.389 to 6.728)	0.517	1.940 (−3.090 to 6.971)	0.449	30.589 (−1.963 to 9.141)	0.205

Linear regression models were used to assess associations between variables

*VMT* verbal memory test, *LSST* letter search speed test, *C-RT* choice reaction time test, *S-RT* simple reaction time test

Significant associations (*p* < 0.05) are highlighted in bold

^a^Indicates log transformed dependent variables

MODEL 1 adjusted for sex, education and childhood cognition; MODEL 2 = MODEL 1 + adjustments for socioeconomic position at age 53, systolic blood pressure and heart rate at age 60–64; MODEL 3 (fully adjusted) = MODEL 2 + adjustments for total cholesterol, smoking, diabetes, diabetes duration and levels of physical activity. In each model, inverse probability weighting was implemented to account for dropout due to death

Numbers in italics indicate statistical significance

When changes of BMI and WC were included in the same fully adjusted model, faster WC gain between 36 and 43 years remained significantly associated with choice reaction time (β = 8.664; 95% CI 2.054 to 15.275; *p* = 0.010), and a weaker association was observed with verbal memory test performance (β = −0.530; 95% CI –1.116 to 0.050; *p* = 0.070). In contrast, the association between gain of WC at 36–43 years with letter search speed as well as between gain of BMI at 36–43 and letter search speed became non-significant.

## Discussion

This study shows that different patterns of whole body and abdominal obesity are associated with cognitive function at 60–64 years. Cumulative exposure to elevated BMI and WC over 30 years was related to poorer memory function at 60–64 years. We identified a sensitive period in early adulthood when a faster gain of BMI and WC might have a greater impact on cognitive capacities in late midlife compared to weight gain in other periods, and show that patterns of cumulative exposure or rapid changes in WC remain associated with cognition even after adjustment for BMI. Finally, we found that the relationships between patterns of adiposity and cognitive function were not explained by CVD risk factors and vascular phenotypes. The process of neurodegeneration leading to cognitive decline and dementia is complex and likely to result from the interaction of multiple factors. Our findings support the adoption of early interventions based on the prevention of central and whole body obesity as possible measures to reduce the burden of cognitive decline in the general population.

The negative association between measures of abdominal and whole body obesity with cognitive function observed in our survey is supported by previous epidemiological and genetic studies [3, 4, 7, 8, 31, 32]. However, only limited data are available on the potential influence of adult patterns of BMI on cognition. Using data from the NSHD cohort, Albanese et al. [24] documented that weight gain during specific periods of life is associated with cognitive capacities at 53 years, although this association was attenuated by socioeconomic position and childhood cognitive capacities. Because our analyses use cognitive function and adiposity measures from a later assessment, comparisons are difficult to make. Albanese et al. [24] also reported results stratified by sex, as significant sex × BMI interactions were identified. In our sample, we found no evidence of effect modification by sex. Our results are broadly consistent with those obtained in the Whitehall II study by Sabia et al. [7], where a dose–response relationship was identified between longer exposure to obesity and lower cognitive function at 60 years old. Similarly, we also provide information on the importance of patterns of WC in addition to BMI for cognition, as well as on the different impact of rapid weight gain at different age of adult life on later cognition. Previous studies exploring the relationship between indices of central adiposity and cognitive function are based on samples of older adults (65+) [6, 33], were cross-sectional [2, 34–36] or had small sample sizes [37]. We are the first to report the influence of cumulative exposure to elevated WC and of rapid changes of WC during adulthood on different cognitive outcomes, and to show that patterns of WC in adulthood could provide additional information in predicting late midlife cognitive functions than patterns of BMI.

In cross-sectional and longitudinal analyses, adjustment for PWV or cIMT had little effect on the relationship between adult patterns of BMI or WC and verbal memory test performance. The influence of cardiovascular factors on the association between whole body and abdominal obesity with cognitive function has been previously explored, albeit with conflicting results [2, 38, 39]. In these studies, cardiovascular risk factors were measured only at a single point, and no measures of subclinical CVD were available. As cIMT and PWV are recognised markers of end-organ damage and reflect the lifetime burden of cardiovascular risk factor exposure, the minimal attenuation of the association between patterns of WC and BMI with memory function after adjustment for PWV and cIMT suggests that obesity and vascular factors might affect cognitive function by different mechanisms and should be treated early and concomitantly to reduce the risk of cognitive impairment. This is supported by results of recent clinical trials, wherein multidomain interventions have been indicated as those likely to represent the most effective strategies to improve cognitive function in overweight populations [40].

Our study has several strengths. The NSHD is the longest-running longitudinal study in the UK, with multiple measures of height, weight and WC available at different ages. It includes individuals without cognitive impairment and is generally representative of the British-born population of similar age. The availability of multiple vascular and cognitive measures enabled exploration of the association between different vascular phenotypes and a wide range of cognitive domains in late mid-life, with appropriate adjustment for environmental and behavioural factors.

Nevertheless, the study also has limitations. First, we examined associations in an observational study and therefore cannot reliably assign causality. Second, the outcome of our analysis was cognitive function and more studies are necessary to test the relevance of our findings against the risk of dementia. Third, attrition is unavoidable in long-running studies such as NSHD, but previous analyses have shown that the samples at 53 and 60–64 years remained broadly representative of the British-born population of that age. Finally, the results in relation to the groups who achieve a stable weight reduction should be interpreted with caution, as only a limited number of participants had sustained weight loss/re-gain during follow-up.

## Conclusion

Increasing cumulative exposure to elevated BMI and WC in adulthood is associated with lower memory function at 60–64 years, and a rapid gain of WC across the third and fourth decades is associated with a global reduction of cognitive capacities in later life. Cardiovascular risk factors and vascular phenotypes are unlikely to account for these associations. Our findings suggest that lifelong prevention of whole body and abdominal obesity, particularly in early midlife, might represent the most effective strategy to prevent the burden of cognitive decline attributable to obesity in the general population.

## Additional file

Additional file 1: Additional Methods and Results (including additional Tables and Figures). (DOC 688 kb)

## Abbreviations

BMI: Body Mass Index
CVD: Cardiovascular Disease
cIMT: common carotid artery Intima-Media Thickness
C-RT: Choice Reaction Time Test
LSST: Letter Search Speed Test
MRC: Medical Research Council
NSHD: National Survey of Health and Development
PWV: Pulse Wave Velocity
S-RT: Simple Reaction Time Test
VMT: Verbal Memory Test
WC: Waist Circumference
