# Supplementary material for: Patterns of adiposity, vascular phenotypes and cognitive function in the 1946 British Birth Cohort
Source: BMC Med. 2018 May 28;16:75. doi: 10.1186/s12916-018-1059-x (PMC5971427; doi:10.1186/s12916-018-1059-x)
Supplement: Supplementary file 1 — Additional Methods and Results (including additional Tables and Figures). (DOC 688 kb) [file 12916_2018_1059_MOESM1_ESM.doc]

**SUPPLEMENTARY**

**PATTERNS OF ADIPOSITY, VASCULAR PHENOTYPES AND COGNITIVE FUNCTION IN THE 1946 BRITISH BIRTH COHORT**

**METHODS**

**Population**

At 60–64 years, participants still alive and with a known current address in mainland Britain (n=2856) were invited for assessment at one of six clinical research facilities (CRF); those unable or unwilling to travel were offered a home visit by a research nurse. A total of 2,229 participants out of the 2856 invited (78.0%) underwent assessment: 1690 attended the CRF and the remaining 539 were seen in their homes. Invitations were not sent to those who had died (n=778), who were living abroad (n=570), had previously withdrawn from the study (n=594) or had been lost to follow-up (n=564). The present study is based on the 1249 (74%) of 1690 participants who attended a clinical research facilities (CRF) and had vascular phenotype and cognitive data at 60-64 years with BMI >18.5 kg/m^2^.

**Cognitive assessment**

The verbal memory test consisted of a 15-item word list learning task. Every participant was shown each word for 2 seconds and then was asked to write down as many of these as possible within 1 minute. This task was repeated twice for a total of three learning trials. Each trial was scored for the total number of different correct words recalled. The sum of the 3 learning trials was used as an outcome measure.

For the letter search speed test, the survey member was asked to cross out as many letters P and W (embedded in a 30 x 20 letter matrix) as quickly and accurately as possible within 1 minute. The participant began at the top of the page and worked from left to right, line by line, without going backwards. The number of targets hit in one minute was used as the final outcome.

Finally, two reaction time tests were performed: simple and choice reaction time. The simple reaction time test required the participant to press one button as quickly as possible following a signal. Eight practice trials were given, followed by 20 ‘real’ trials. To avoid anticipation there was a variable delay (1-3 sec) between each response and the next signal. The outcome measure was calculated as the average reaction time across the 20 trials (in seconds). The choice reaction time test required survey members to press one of four buttons as quickly as possible corresponding to which of the numbers 1 to 4 appeared on the signal screen. Eight practice trials were given, followed by 40 ‘real’ trials. A similar variable delay between numbers to simple reaction time test was used to avoid anticipation. The average reaction time of the correct trials was used as the outcome measure.

**Vascular Phenotypes measured at age 60-64 years**

*Carotid to femoral pulse wave velocity (PWV)*

Participants were rested in a supine position for 10 min before the measurement in a quiet room. Measurements of PWV were obtained using a Vicorder device by placing a 10-cm-wide cuff around the right upper thigh to detect the femoral pulse and a 3-cm cuff around the neck with the pressure pad over the right carotid area to detect the right carotid pulse. Path length was defined as the distance from the suprasternal notch to the middle of the thigh cuff minus the distance from the suprasternal notch to the neck cuff; the thigh cuff modification was in accordance with a recent validation study. The cuffs were inflated simultaneously to 65mmHg and high quality pulse waveforms were recorded for 10s. Foot-to-foot transit time was determined using an in-built cross-correlation algorithm and final values of PWV were both recorded by nurses on the participant resultsheet and exported using an excel format onto an external encrypted hard drive.

*Common Carotid Intima-Media Thickness (cIMT)*

Both the left and right common carotid arteries were imaged longitudinally 1 cm proximal to the carotid bifurcation using an ultrasound scanner (Vivid I, GE Healthcare) with a high resolution probe (12MHz) and following a standardized protocol. Once clear images were obtained, the zoom function was used to magnify the region of interest. Ten second cineloops were recorded in DICOM format and downloaded for offline analysis. Analysis of the cineloops was performed in a core laboratory (Vascular Physiology Unit, Institute of Cardiovascular Science, University College of London) using dedicated software (Carotid Analyser, Iowa City, Iowa), which allows semi-automatic edge detection of the echogenic lines of the intima–media complex. Three end-diastolic frames were selected and analysed to determine the mean cIMT of each image, defined as the interface between lumen-intima and media-adventitia of the far wall of the common carotid artery. All images were analysed by two trained readers who were blinded to participant identity and characteristics. The average of right and left cIMT was used in all analyses. Intra- and inter-reader reproducibility were evaluated using a subset of 10 randomly selected images where each image was analysed by each reader twice. Intraclass correlation coefficients were >0.9 for both within and between reader comparisons.

**Covariates**

Blood pressure and heart rate were recorded at the same clinic visit as the cardiovascular phenotype assessment (60–64 years). Blood pressure was measured with the participant in a seated position twice, and the second readings were used in all analyses. When two valid readings were not available, we used the single reading. The highest educational qualification achieved by age 26 years was dichotomized into those with advanced (’A level’, taken during the final year of secondary/high school) or higher (university or equivalent) qualifications, versus those below this level. Childhood cognitive ability was measured using a series of cognitive tests when participants were 8 years old by teachers or trained cares, as previously described. Occupational social class at age 53 was used as an indicator of socioeconomic position in adulthood and categorized using the Registrar General's Social Classification into 6 groups, from unskilled to professional occupations. We used a range of measures at 60-64 years to characterize cardiovascular risk. Smoking status was obtained from self-reported questionnaires and participants were classified as never, past or current smokers. The medications used by each participant were recorded by study nurses who also collected overnight fasting blood samples to measure total cholesterol, HDL cholesterol, and triglycerides, as previously described. HbA1c was measured with a Tosoh A1c 2.2 analyser. C-reactive protein was measured colorimetrically with a Siemens Dimension Xpandanalyser, while concentrations of leptin and adiponectin were measured with radioimmunoassay and ELISA (R&D systems, Abingdon, UK), respectively. Diabetes mellitus at age 60–64, coded as a binary variable, was a self-reported diagnosis, or glycated haemoglobin of >6.5%, or reported use of prescribed insulin or oral antidiabetic agents. Duration of diabetes and levels of physical activity in the last 4 weeks were self-reported. Levels of physical activity were categorized as none, moderate or intense. History of CVD was derived from self-reported doctor diagnosed nonfatal myocardial infarction, acute coronary syndrome, surgical and percutaneous coronary revascularization, angina pectoris, chronic ischemic heart disease, stroke and coded as a binary variable.

**Statistical analysis**

For descriptive analyses, *mean ± standard deviation (SD)* are presented for continuous variables and percentage for binary variables. In cross-sectional analyses, we made comparisons between individuals who were normal weight, overweight, and obese at age 60-64 years for cardiovascular risk factors, cIMT, PWV and cognitive outcomes (verbal memory, letter search speed, choice and simple reaction time tests) using linear regression with a test for linear trend carried out across the three categories. We tested for effect modification of obesity indices on cognitive outcomes according to gender by introducing relevant interaction terms (BMI*sex) in multivariable regression models. Level of statistical significance for interaction terms was set at 0.1 and when a significant interaction was found, results were stratified by sex. We then fitted a series of multivariable regression models to establish the cross-sectional associations of BMI (as a continuous variable) with each cognitive outcome and with each vascular phenotype (PWV, cIMT). In each model, inverse probability weighting was implemented to appropriately account for drop out due to death. Weights were derived from regressing the outcome of death by the 60-64 year visit (yes or no) on different combinations of exposure variables at the baseline in the full sample. All significant (p<0.05) co-variates as well as biologically plausible variables (i.e. gender) were included in the models. The models were sequentially adjusted for factors associated with cognitive measures in cross-sectional analyses or in previous reports as follows: MODEL 1 adjusted for sex, education and childhood cognition; MODEL 2 = MODEL 1 + socio-economic position at 53 years, systolic blood pressure and heart rate at age 60-64; MODEL 3 (fully adjusted) = MODEL 2 + total cholesterol, smoking, diabetes and levels of physical activity. We then assessed the associations between each vascular phenotype (exposure) and each cognitive outcome using the same series of multivariable models. Brain damage and memory decline related to increased PWV is considered to be mediated, at least in part, by increased pulse pressure (PP = systolic blood pressure – diastolic blood pressure). Therefore, for associations found to be significant at the 5% level, we also assessed whether inclusion of PP rather than systolic blood pressure in MODEL 2 and 3 materially affected any association between vascular phenotypes and cognitive outcomes.

To assess the proportion of the association between BMI and each cognitive test explained by vascular phenotypes, we added each of PWV and cIMT in turn to the fully adjusted model (MODEL 3) and considered the percentage change of the regression coefficients for obesity parameters as an estimate of the potential mediated effect of obesity on cognitive outcomes through adverse vascular phenotypes. Each analysis was repeated using waist circumference (WC) rather than BMI as exposure. Finally, to assess whether central adiposity had an effect over and above the effect of general adiposity, each cross-sectional and longitudinal analysis was repeated including BMI and WC in the same models.

Study members with vascular phenotypes at 60-64 years and complete BMI records at all ages were included in longitudinal analyses. To test the possible influence of duration of obesity from age 36 on cognitive function at age 60-64 years, we grouped individuals according to seven different patterns of BMI change: (a) those who maintained a normal weight up to 60-64 years; (b) those who were overweight/obese at all ages; groups c–e included individuals who maintained overweight/obese status from age 43, 53, or 60–64 years, respectively; (f) those who were overweight/obese at some point, but who dropped a category (from obese to overweight or overweight to normal) and did not regain; and (g) those who were overweight/obese at some point and who dropped but subsequently regained a category. We investigated differences in each cognitive outcome (verbal memory, letter search speed, choice and simple reaction time tests) across the four ages at first overweight/ obese groups (b–e) using a test for trend, with participants in the never overweight/obese (group a) used as the reference. Additionally, all categories were compared to the reference group (a) for each cognitive outcome. The same approach to adjustment was used as for the cross-sectional analyses (Models 1, 2 and 3), but MODEL 3 was further adjusted for duration of diabetes. To assess the extent of the association between cumulative overweight/obesity and cognitive tests explained by vascular phenotypes, we added each vascular phenotype variable (PWV and cIMT) in turn to the fully adjusted model. Finally, we tested for effect modification by socio-economic position and education level of the association between obesity indices on cognitive outcomes. Interaction terms (i.e. changes in BMI at different periods*SES and changes in BMI between different periods*education level) were entered into the final adjusted models (Model 3) and considered significant at p<0.1. In case the interaction term or few individual categories of the exposure variable socio-economic position (SES) presented an interaction with BMI/WC changes towards cognition indices, a likelihood ratio test was implemented to assess the improvement of fit of the unrestricted model (with the interaction term) upon adding the interaction term in the restricted model (i.e. without the interaction term). Each analysis was repeated using waist circumference as exposure. In accordance with the WHO classification of the cardio-metabolic risk related to WC, the following groups were identified in our sample: a) Low risk = WC ≤94 cm for males and ≤80 cm for females; b) Increased risk = WC >94 cm and ≤102 cm for males and >80 cm and ≤88 cm for females; c) Substantially increased risk = >102 cm for males and >88 cm for females. Participants in the classes defined as increased and substantially increased risk were combined into an elevated WC group. Thus, we created the same groups used for BMI and performed the same analysis to assess whether patterns of exposure to elevated waist circumference were associated with cognitive outcomes. To explore whether categories of WC had an effect over and above the effect of categories of BMI on each cognitive outcome, we included categories of BMI and WC in the same fully adjusted model.

To investigate whether the rate of change of BMI at a particular period of midlife was more strongly associated with cognitive function, we calculated the conditional change in BMI for different periods of life. We calculated the change in BMI for the periods 36–43 years, 43–53 years, and 53–60/64 years conditional on earlier BMI by modelling each BMI measure (from 43 years onwards) on the earlier measure(s) and saving the residuals. These residuals reflect the mean rate of change in BMI and can be interpreted as the change in BMI above or below that expected on average, given earlier BMI. The residuals were standardised to allow a comparison of the relative strength of associations between periods. We subsequently fitted regression models including all these standardised changes with each cognitive function as outcomes (verbal memory, letter search speed, choice and simple reaction time tests). Models were progressively adjusted as described in the cross-sectional analysis. Using the same approach, we estimated, standardised, and regressed residuals of waist circumference against each cognitive outcome, following the same steps for adjustment as described in the cross-sectional analysis. Finally, to explore whether changes of WC at each period had an effect over and above the effect of changes of BMI on each cognitive outcome, residuals of BMI and WC were included in the same fully adjusted model.

To test whether the association between overweight/obese or WC groups and cognitive tests differed by blood pressure medication use we also ran the models with an interaction between medication use and overweight/obese groups. In sensitivity analyses, we restricted our sample to those without a previous history of CVD. Statistical analyses were performed with STATA (version 13.1) software.

**RESULTS**

**Table 1S.** Associations between BMI and letter search speed test at age 60-64, stratified by sex.

|  | **MODEL 1** | | **MODEL 2** | | **MODEL 3** | |
| --- | --- | --- | --- | --- | --- | --- |
|  | **β (95% CI)** | **P** | **β (95% CI)** | **P** | **β (95% CI)** | **P** |
| **Males** | -0.003 (-0.011; 0.010) | 0.948 | -0.002 (-0.012; 0.009) | 0.761 | -0.0002 (-0.012; 0.012) | 0.978 |
| **Females** | -0.007 (-0.012; -0.003) | **0.001** | -0.006 (-0.011; -0.001) | **0.011** | -0.006 (-0.011; -0.0004) | **0.036** |

Linear regression models were used to assess associations between variables. LSST was log transformed. MODEL 1 adjusted for education and childhood cognition; MODEL 2 = MODEL 1 + adjustments for socio-economic position at age 53, systolic blood pressure and heart rate at age 60-64; MODEL 3 (fully adjusted) = MODEL 2 + adjustments for total cholesterol, smoking, diabetes and levels of physical activity. In each model, inverse probability weighting was implemented to account for drop out due to death. Associations achieving statistical significance at P<0.05 are highlighted in bold.

**Table 2S.** Cross-sectional associations of waist circumference with cIMT, PWV and verbal memory, letter search speed, choice and simple reaction time tests at age 60-64 years.

|  | **Model 1** | | **Model 2** | | **Model 3** | |
| --- | --- | --- | --- | --- | --- | --- |
|  | **β (95% CI)** | **P** | **β (95% CI)** | **P** | **β (95% CI)** | **P** |
| **VASCULAR MEASURES** | | | | | | |
| **1. cIMT (mm)** | 0.001 (0.00004; 0.002) | **0.040** | 0.001 (-0.0002; 0.001) | 0.140 | 0.001 (0.0001; 0.002) | **0.039** |
| **2. PWV (m/s)** | 0.015 (0.004;0.026) | **0.006** | 0.006 (-0.004; 0.017) | 0.226 | 0.005 (-0.006; 0.015) | 0.375 |
| **COGNITIVE MEASURES** | | | | | | |
| **1. VMT (n. of words)** | -0.073 (-0.104; -0.041) | **<0.001** | -0.065 (-0.097; -0.033) | **<0.001** | -0.062 (-0.096: -0.028) | **<0.001** |
| **2. LSST* (targets)** | -0.001 (-0.003; 0.001) | 0.460 | -0.001 (-0.002; 0.001) | 0.591 | -0.0001 (-0.002; 0.002) | 0.864 |
| **3. RT (s)** |  | | | | | |
| **a. Simple** | 0.191 (-0.134; 0.512) | 0.249 | 0.219 (-0.114; 0.553) | 0.197 | 0.275 (-0.097; 0.649) | 0.147 |
| **b. Choice** | 0.607 (0.171; 1.044) | **0.006** | 0.648 (0.210; 1.086) | **0.004** | 0.640 (0.152; 1.129) | **0.010** |

Linear regression models were used to assess associations between variables.

MODEL 1 adjusted for sex, education, childhood cognition; MODEL 2 = MODEL 1 + adjustments for socio-economic position at age 53, systolic blood pressure and heart rate at age 60-64; MODEL 3 (fully adjusted) = MODEL 2 + adjustments for total cholesterol, smoking, diabetes, duration of diabetes and levels of physical activity. In each model, inverse probability weighting was implemented to account for drop out due to death.

cIMT = Common Carotid Artery Intima-Media Thickness. PWV = Pulse Wave Velocity. VMT = Verbal Memory Test. LSST = Letter Search Speed Test. RT = Reaction time test.

*indicates log transformed dependent variables

**Table 3S. Association between cardiovascular risk factors and cognitive tests.**

|  | **Verbal memory test (number of words)** | | **Letter search speed test*** | | **Simple reaction time (s)** | | **Choice reaction time (s)** | |
| --- | --- | --- | --- | --- | --- | --- | --- | --- |
|  | **β (95% CI)** | **P** | **β (95% CI)** | **P** | **β (95% CI)** | **P** | **β (95% CI)** | **P** |
| **Total Cholesterol (mmol/L)** | 0.013 (-0.002, 0.028) | 0.083 | 0.0001(-0.0003, 0.0006) | 0.574 | -0.001(-0.002, 0.0002) | 0.104 | -0.001(-0.002, 8.19x10^-06^) | 0.052 |
| **LDL-Cholesterol (mmol/L)** | 0.009 (-0.004, 0.022) | 0.177 | 0.0002(-0.0002, 0.0006) | 0.374 | -0.0008 (-0.002, 0.0004) | 0.204 | -0.001(-0.002, 0.0003) | 0.192 |
| **HDL-Cholesterol (mmol/L)** | 0.005 (-0.0004, 0.009) | 0.070 | -0.0001(-0.0002, 0.0001) | 0.586 | -0.0003(-0.0007, 0.0001) | 0.261 | -0.0004 (-0.001, -0.0001) | **0.022** |
| **Triglycerides(mmol/L)*** | -0.005 (-0.012, 0.002) | 0.141 | -4.25x10^-7^ (-0.0002, 0.0002) | 0.997 | -0.0002(-0.001, 0.0004) | 0.558 | 0.0002 (-0.0003, 0.001) | 0.485 |
| **Heart Rate (bpm)** | -0.144 (-0.290, 0.003) | 0.055 | -0.005(-0.010, -0.0001) | **0.042** | 0.0004(-0.014, 0.013) | 0.950 | -0.011(-0.029, 0.006) | 0.887 |
| **Systolic Blood Pressure (mmHg)** | -0.143 (-0.373, 0.086) | 0.221 | -0.004(-0.008, 0.007) | 0.901 | -0.017(-0.038, 0.004) | 0.103 | 0.011(-0.029, 0.005) | 0.186 |
| **Diastolic Blood Pressure (mmHg)** | -0.068 (-0.191, 0.055) | 0.279 | 0.001(-0.003, 0.005) | 0.609 | -0.014(-0.025, -0.003) | **0.014** | -0.011(-0.020, -0.002) | 0.021 |
| **Diabetes** | 0.0004 (-0.005, 0.006) | 0.894 | -0.0001(-0.0004, 0.0001) | 0.249 | 0.0001(-0.0006, 0.0005) | 0.626 | -0.0002(-0.001, 0.0002) | 0.228 |
| **Current Smoking** | -0.004 (-0.011, 0.002) | 0.176 | 0.0001(-0.0001, 0.0003) | 0.359 | -0.0001(-0.0007, 0.0005) | 0.713 | 0.001(0.00004, 0.001) | **0.033** |
| **C-reactive protein (mg/L)*** | -0.002 (-0.015, 0.010) | 0.718 | -0.00007(-0.0004, 0.0002) | 0.721 | 0.001 (0.0003, 0.002) | **0.011** | 0.001(3.33x10^-06^, 0.001) | **0.049** |
| **Physical activity level (@60-64)** | 0.015 (0.004, 0.027) | **0.011** | 0.0003(-0.00008, 0.0007) | 0.117 | -0.0003 (-.001, 0.0007) | 0.491 | -0.0001 (-0.001, 0.001) | 0.660 |

Linear regression models were used to assess associations between variables. *Log transformed variable. All analyses adjusted for sex, education and childhood cognition. Significant associations are highlighted in bold.

**Table 4S. Characteristics of the sample at 60-64 years by patterns of adult overweight/obesity.**

|  | **Overweight/**  **Obese**  **@36** | **Overweight/**  **Obese**  **@43** | **Overweight/**  **Obese**  **@53** | **Overweight/**  **Obese**  **@60-64** | **Normal**  **weight** | **P-trend** | **Weight loss/**  **No regain** | **Weight loss/**  **Regain** |
| --- | --- | --- | --- | --- | --- | --- | --- | --- |
| **N** | 223 | 147 | 180 | 92 | 238 |  | 63 | 78 |
| Education (% above A-level) | 58% | 63% | 69% | 53% | 65% | 0.133 | 55% | 50% |
| Systolic blood pressure (mmHg) | 138.7±18.3 | 136.5±18.7 | 135.7±18.2 | 134.7±17 | 132.7±18.2 | **<0.001** | 133.7±16.9 | 136.3±15.4 |
| Diastolic blood pressure (mmHg) | 79±10.3 | 78±9.4 | 78.2±9.8 | 76.8±8.5 | 74±9.1 | **<0.001** | 76.3±9.5 | 77.9±8.5 |
| Total-cholesterol (mmol/L) | 5.3±1.3 | 5.7±1.1 | 5.7±1.3 | 6.1±1.1 | 5.8±1.1 | **0.026** | 5.7±1.2 | 5.6±1.2 |
| LDL-cholesterol (mmol/L) | 3.2±1.1 | 3.6±1.0 | 3.5±1.1 | 3.8±1.0 | 3.6±0.9 | 0.085 | 3.6±1.0 | 3.5±1.0 |
| HDL-cholesterol (mmol/L) | 1.4±0.3 | 1.5±0.3 | 1.6±0.4 | 1.7±0.4 | 1.8+0.4 | **<0.001** | 1.6±0.3 | 1.5±0.4 |
| Triglycerides* (mmol/L) | 1.3[1.0-1.8] | 1.3[0.9-1.8] | 1.1[0.8-1.5] | 1.0[0.8-1.6] | 0.8[0.6-1.2] | **<0.001** | 0.9[0.7-1.1] | 1.2[1.0-1.6] |
| HBA1C* (%) | 40[37-44] | 39[37-42] | 40[38-42] | 40[37-42] | 39[37-41] | 0.12 | 39[37-41] | 40[37-43] |
| Adiponectin* (μg/ml) | 9.7[6.0-14.5] | 12.5[7.7-17.7] | 12.6[7.5-18.4] | 14.7[8.4-21.2] | 16.7[11.1-25.1] | **<0.001** | 12.2[8.2-23.2] | 10.5[6.3-17.2] |
| Leptin* (ng/ml) | 18.1[10.4-34.6] | 16.1[7.4-32.8] | 17.2[8.7-29.7] | 12.1[6.2-22.2] | 7.3[4-12] | **<0.001** | 8.6[3.3-14.1] | 12.2[7.3-24.5] |
| Pulse Wave Velocity (m/s) | 8.39±1.48 | 8.35±1.37 | 8.17±1.56 | 8.17±1.54 | 7.9±1.51 | **0.001** | 8.12±1.61 | 8.38±1.49 |
| Carotid Intima-Media Thickness (mm) | 0.73±0.14 | 0.71±0.13 | 0.69±0.12 | 0.68±0.14 | 0.67±0.11 | **<0.001** | 0.66±0.11 | 0.68±0.13 |

Values are presented as mean ± standard deviation, N (%) or *median [IQR]. Comparisons between groups were performed using linear regression model.

**Table 5S.** Characteristics of the sample at 60-64 years by patterns of elevated waist circumference (HiWC).

|  | **HiWC**  **@36** | **HiWC**  **@43** | **HiWC**  **@53** | **HiWC**  **@60-64** | **Normal**  **waist** | **P-trend** | **Waist loss/**  **No regain** | **Waist loss/**  **Regain** |
| --- | --- | --- | --- | --- | --- | --- | --- | --- |
| Education (% above A-level) | 55% | 61% | 62% | 54% | 76% | **0.025** | 56% | 57% |
| Systolic blood pressure (mmHg) | 140±15.5 | 137.6±201 | 135±16.9 | 133±18.7 | 132±16.8 | **<0.001** | 129±17.4 | 137±18.4 |
| Diastolic blood pressure (mmHg) | 79±10.2 | 78±10.9 | 78±9.3 | 76±8.9 | 75±9.6 | **0.001** | 74±9.6 | 78±9.2 |
| Total-cholesterol (mmol/L) | 5.14±1.25 | 5.58±1.28 | 5.67±1.26 | 6.06±1.21 | 5.76±1.02 | **<0.001** | 5.66±1.06 | 5.78±1.09 |
| LDL-cholesterol (mmol/L) | 3.08±1.05 | 3.44±1.12 | 3.52±1.10 | 3.77±1.06 | 3.58±0.87 | **<0.001** | 3.46±0.88 | 3.61±0.92 |
| HDL-cholesterol (mmol/L) | 1.39±0.32 | 1.47±0.35 | 1.58±0.36 | 1.74±0.45 | 1.76±0.419 | **<0.001** | 1.72±0.42 | 1.56±0.36 |
| Triglycerides* (mmol/L) | 1.3[0.99-1.91] | 1.27[0.97-1.97] | 1.06 [0.79-1.42] | 1.03 [0.73-1.55] | 0.83 [0.63-1.12] | **<0.001** | 0.89 [0.69-1.12] | 1.21[0.94-1.62] |
| HBA1C* (%) | 41[37.5-44] | 39 [37-43] | 39 [37-42] | 39.5 [37-41] | 39 [37-41] | **<0.001** | 40 [38-41] | 40[38-42] |
| Adiponectin* (μg/ml) | 10.2[5.98-15.5] | 10.4 [6.41-16.39] | 13.25 [7.55-19.31] | 13.25 [8.52-21.42] | 14.74 [8.95-24.71] | **<0.001** | 15.54[11.63-24.54] | 11.64[7.45-17.75] |
| Leptin* (ng/ml) | 23.2[12-38.7] | 15[9-34.2] | 16.15[8.05-29.65] | 11.95[6.8-19.7] | 5.25[3.15-8.45] | **<0.001** | 7.6[4.3-10.5] | 15.2[10.5-24-8] |
| Pulse Wave Velocity (m/s) | 8.74±1.59 | 8.35±1.44 | 8.24±1.46 | 8.01± 1.70 | 7.87±1.42 | **<0.001** | 8.02±1.28 | 8.26±1.34 |
| Carotid Intima-Media Thickness (mm) | 0.73±0.14 | 0.71± 0.13 | 0.70±0.12 | 0.67±0.13 | 0.66±0.11 | **<0.001** | 0.68±0.13 | 0.71±0.12 |

Values are presented as mean ± standard deviation, N (%) or *median [IQR]. Comparisons between groups were performed using linear regression model.

**Table 6S. Relationship between cardiovascular risk factors and phenotypes at age 60–64 years with rate of change in BMI at three time periods: BMI_36-43_ = rate of BMI increase 36–43 years, BMI_43-53_ = rate of BMI increase 43–53 years, and BMI_53-60 to 64_ = rate of BMI increase 53–60 to 64 years.**

|  | **BMI_36-43_** | | **BMI_43-53_** | | **BMI_53-60 to 64_** | |
| --- | --- | --- | --- | --- | --- | --- |
|  | **β (95% CI)** | **P** | **β (95% CI)** | **P** | **β (95% CI)** | **P** |
| Education (% above A-level) | -0.173(-0.316; -0.031) | 0.017 | 0.068 (-0.070; 0.206) | 0.333 | -0.074 (-0.213; 0.064) | 0.294 |
| Systolic blood pressure (mmHg) | 0.588(-0.468; 1.644) | 0.275 | 0.877 (-0.164; 1.918) | 0.099 | 1.85 (0.832; 2.874) | **<0.001** |
| Diastolic blood pressure (mmHg) | 0.165(-0.411; 0.741) | 0.574 | 0.618 (0.058; 1.178) | **0.03** | 1.01 (0.459; 1.568) | **<0.001** |
| Heart Rate (bpm) | 0.936(0.274; 1.598) | **0.006** | 1.05 (0.395; 1.710) | **0.002** | 0.677 (0.032; 1.321) | **0.04** |
| Total-cholesterol (mmol/L) | -0.023(-0.097; 0.051) | 0.543 | -0.026 (-0.097; 0.045) | 0.475 | 0.039 (-0.031; 0.108) | 0.277 |
| LDL-cholesterol (mmol/L) | -0.031(-0.095; 0.033) | 0.339 | -0.019 (-0.081; 0.042) | 0.537 | 0.034 (-0.027; 0.094) | 0.273 |
| HDL-cholesterol (mmol/L) | -0.02(-0.045; 0.005) | 0.119 | -0.049 (-0.073; -0.024) | **<0.001** | -0.05 (-0.074; -0.026) | **<0.001** |
| Triglycerides* (mmol/L) | 0.036(0.006; 0.067) | **0.021** | 0.081 (0.051; 0.110) | **<0.001** | 0.101 (0.072; 0.131) | **<0.001** |
| HBA1C* (%) | 0.006(-0.003; 0.015) | 0.163 | 0.026 (0.018; 0.035) | **<0.001** | 0.001 (-0.007; 0.009) | 0.790 |
| C-reactive protein (mg/L)* | 0.019(-0.037; 0.076) | 0.499 | 0.068 (0.012; 0.124) | **0.017** | 0.071 (0.020; 0.123) | **0.006** |
| Adiponectin* (μg/ml) | -0.01(-0.048; 0.038) | 0.822 | -0.052 (-0.094; -0.010) | **0.016** | -0.054 (-0.096; -0.013) | **0.011** |
| Leptin* (ng/ml) | 0.268(0.212; 0.323) | **<0.001** | 0.322 (0.270; 0.374) | **<0.001** | 0.36 (0.309; 0.410) | **<0.001** |
| PWV (m/s) | 0.095(0.006; 0.183) | **0.036** | 0.045 (-0.044; 0.133) | 0.321 | 0.061 (-0.026; 0.149) | 0.170 |
| cIMT (mm) | 0.002(-0.007; 0.011) | 0.645 | 0.002(-0.007; 0.011) | 0.698 | 0.01 (-0.0004; 0.017) | 0.063 |

Linear regression models were used to assess associations between variables. All analyses are unadjusted.

*Indicates log transformed dependent variables. Significant associations are highlighted in bold.

**Table 7S. Relationship between cardiovascular risk factors and phenotypes at age 60–64 years with rate of change in waist circumference (Waist) at three time periods: Waist_36-43_ = rate of waist circumference increase 36–43 years, Waist_43-53_ = rate of waist circumference increase 43–53 years, and Waist_53-60 to 64_ = rate of waist circumference increase 53–60 to 64 years.**

|  | **Waist_36-43_** | | **Waist_43-53_** | | **Waist_53-60 to 64_** | |
| --- | --- | --- | --- | --- | --- | --- |
|  | **β (95% CI)** | **P** | **β (95% CI)** | **P** | **β (95% CI)** | **P** |
| Education (% above A-level) |  |  |  |  |  |  |
| Systolic blood pressure (mmHg) | 2.330 (1.286, 3.375) | **<0.001** | 1.204 (.174, 2.236) | **0.022** | 1.924 (0.911, 2.938) | **<0.001** |
| Diastolic blood pressure (mmHg) | 1.104 (0.537, 1.671) | **<0.001** | 0.893 (0.338, 1.447) | **0.002** | 0.857 (0.306, 1.408) | **0.002** |
| Heart Rate (bpm) | 0.037 (-0.623, 0.698) | 0.912 | 0.866 (.214, 1.518) | **0.009** | 1.053 (0.415, 1.691) | **0.001** |
| Total-cholesterol (mmol/L) | -0.217 (-0.289, -0.144) | **<0.001** | -0.069 (-0.140, 0.002) | 0.056 | 0.050 (-0.019, 0.119) | 0.157 |
| LDL-cholesterol (mmol/L) | -0.164 (-0.226, -0.102) | **<0.001** | -0.047 (-0.108, 0.014) | 0.134 | 0.037 (-0.022, 0.097) | 0.220 |
| HDL-cholesterol (mmol/L) | -0.105 (-0.129, -0.081) | **<0.001** | -0.067 (-0.091, -0.042) | **<0.001** | -0.053 (-0.077, -0.029) | **<0.001** |
| Triglycerides* (mmol/L) | 0.085 (0.055, 0.115) | **<0.001** | 0.090 (0.060, 0.120) | **<0.001** | 0.107 (0.078, 0.136) | **<0.001** |
| HBA1C* (%) | 0.014 (0.006, 0.023) | **0.001** | 0.019 (0.011, 0.028) | **<0.001** | 0.013 (0.005, 0.021) | **0.002** |
| C-reactive protein (mg/L)* | 0.053 (-0.001, 0.108) | 0.055 | 0.087 (0.032, 0.142) | **0.002** | 0.080 (.0277025 .1329) | **0.003** |
| Adiponectin* (μg/ml) | -0.178 (-0.219, -0.137) | **<0.001** | -0.065 (-0.107, -0.023) | **0.002** | -0.068 (-0.109, -0.027) | **<0.001** |
| Leptin* (ng/ml) | 2.525 (2.473, 2.578) | **<0.001** | 2.525 (2.473, 2.578) | **<0.001** | 2.525 (2.473, 2.578) | **<0.001** |
| PWV (m/s) | 0.126 (0.038, 0.215) | **0.005** | 0.117 (0.030, 0.204) | **0.009** | 0.072 (-0.014, 0.159) | 0.102 |
| cIMT (mm) | 0.016 (0.007, 0.024) | **0.001** | -0.0002 (-0.009, 0.008) | 0.959 | 0.006 (-0.002, 0.014) | 0.164 |

Linear regression models were used to assess associations between variables. All analyses are unadjusted.

*Indicates log transformed dependent variables. Significant associations are highlighted in bold.

**Table 8S**. Relationship stratified by sex between LSST at age 60–64 years and residual changes in BMI between three time periods: BMI_36-43_= rate of BMI increase 36–43 years, BMI_43-53_= rate of BMI increase 43–53 years, and BMI_53-60_= rate of BMI increase 53–60 to 64 years.

|  | **MODEL 1** | | **MODEL 2** | | **MODEL 3** | |
| --- | --- | --- | --- | --- | --- | --- |
|  | **β (95% CI)** | **P** | **β (95% CI)** | **P** | **β (95% CI)** | **P** |
| **Females** |  |  |  |  |  |  |
| **BMI_36-43_** | -0.029 (-0.055, -0.003) | **0.029** | -0.027 (-0.053, -0.001) | **0.041** | -0.027 (-0.055, 0.0003) | **0.052** |
| **BMI_43-53_** | -0.021 (-0.047, 0.004) | 0.106 | -0.017 (-0.042, 0.009) | 0.193 | -0.015 (-0.041, 0.011) | 0.251 |
| **BMI_53-60 to 64_** | -0.003 (-0.030, 0.023) | 0.806 | -0.004 (-0.031, 0.022) | 0.744 | 0.006 (-0.028, 0.039) | 0.741 |
| **Males** |  |  |  |  |  |  |
| **BMI_36-43_** | -0.012 (-0.049, 0.025) | 0.511 | -0.013 (-0.051, 0.024) | 0.483 | -0.025 (-0.065, 0.014) | 0.208 |
| **BMI_43-53_** | 0.005 (-0.034, 0.043) | 0.811 | 0.003 (-0.036, 0.043) | 0.875 | 0.010 (-0.033, 0.052) | 0.647 |
| **BMI_53-60 to 64_** | 0.020 (-0.027, 0.067) | 0.413 | 0.017 (-0.030, 0.065) | 0.476 | 0.028 (-0.026, 0.083) | 0.308 |

Linear regression models were used to assess associations between variables.

MODEL 1 adjusted for education and childhood cognition; MODEL 2 = MODEL 1 + adjustments for socio-economic position at age 53, systolic blood pressure and heart rate at age 60-64; MODEL 3 (fully adjusted) = MODEL 2 + adjustments for total cholesterol, smoking, diabetes, duration of diabetes and levels of physical activity. In each model, inverse probability weighting was implemented to account for drop out due to death.

Significant associations (p<0.05) are highlighted in bold.

**Table 9S. Associations between BMI changes between 36-43 years with choice reaction time (C-RT) stratified by socio-economic position.** Six categories of socio-economic position were available: I. professional (n=116 in our sample), II. intermediate (n=549 in our sample), IIINM. skilled non-manual (n=303), IIIM. skilled manual (n=152), IV. partially skilled (n=100) and V. unskilled (n=23). Due to the low number of survey participants, categories IV and V were merged for the analyses.

| **Categories of socio-economic position** | **MODEL 1** | | **MODEL 2** | | **MODEL 3** | |
| --- | --- | --- | --- | --- | --- | --- |
|  | **β (95% CI)** | **P** | **β (95% CI)** | **P** | **β (95% CI)** | **P** |
| I | 35.232 (6.947, 63.516) | **0.015** | 28.742 (-3.527, 61.011) | 0.080 | 45.289 (5.321, 85.258) | **0.027** |
| II | 13.485 (-1.013, 27.983) | 0.068 | 13.958 (-0.9328, 28.849) | 0.066 | 14.893 (-1.621, 31.406) | 0.077 |
| IIINM | 4.259 (-24.813, 33.332) | 0.773 | 4.816 (-25.608, 35.240) | 0.755 | 9.721 (-23.157, 42.600) | 0.560 |
| IIIM | 1.453 (-44.725, 47.632) | 0.950 | 4.991 (-41.464, 51.447) | 0.830 | -2.048 (-51.626, 47.530) | 0.934 |
| IV+V | -18.311 (-59.136, 22.513) | 0.370 | -17.249 (-58.823, 24.324) | 0.406 | 17.236 (-61.127, 26.654) | 0.428 |

Linear regression models were used to assess associations between variables.

MODEL 1 adjusted for education and childhood cognition; MODEL 2 = MODEL 1 + adjustments for socio-economic position at age 53, systolic blood pressure and heart rate at age 60-64; MODEL 3 (fully adjusted) = MODEL 2 + adjustments for total cholesterol, smoking, diabetes, duration of diabetes and levels of physical activity. In each model, inverse probability weighting was implemented to account for drop out due to death.

Significant associations (p<0.05) are highlighted in bold.

**Figure 1S. Relationship of categories of overweight/obesity change and letter search speed test (LSST) result at age 60–64 years in the whole population (A) and in females alone (B).** Data points are log number of targets hit and bars represent 95% CI for each group. O/O@36, O/O@43, O/O@53, O/O@60-64 = Overweight/obesity since 36, 43, 53 and 60-64 years old, respectively; Never O/O = never overweight/obese; Lost/Non regain = dropped and did not regained one category of BMI; Lost/Regain = dropped and regained one category of BMI. Results are adjusted for sex, childhood cognition, education and inverse probability weighting was implemented to account for drop out due to death.

**A)**

**
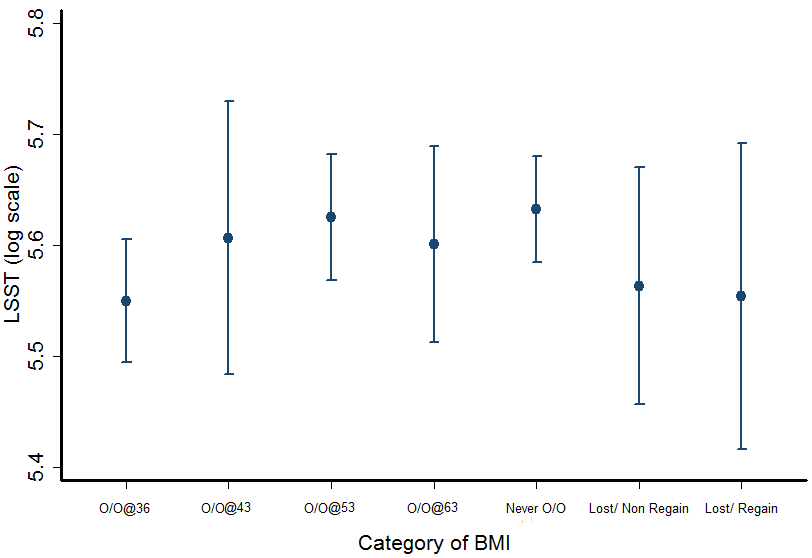
**

**B)**


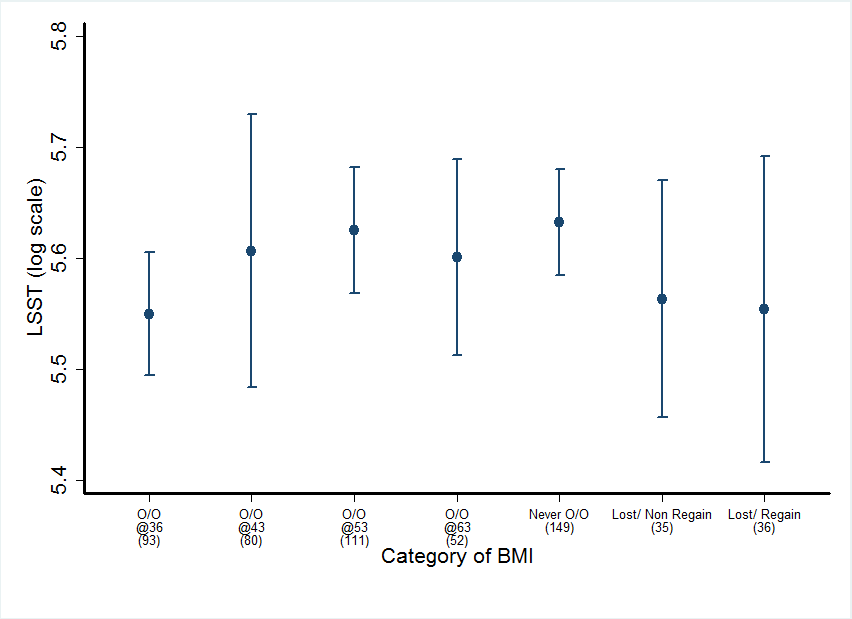


**Figure 2S. Patterns of overweight/obesity change and performance at the simple (A) and choice (B) reaction time tests at age 60–64 years.** Data points are mean reaction time in seconds and bars represent 95% CI for each group. O/O@36, O/O@43, O/O@53, O/O@60-64 = Overweight/obesity since 36,43, 53 and 60-64 years old, respectively; Never O/O= never overweight/obese; Lost/Non regain = dropped and did not regained one category of BMI; Lost/Regain = dropped and regained one category of BMI. Results are adjusted for sex, childhood cognition, education and inverse probability weighting was implemented to account for drop out due to death.

**A) B)**


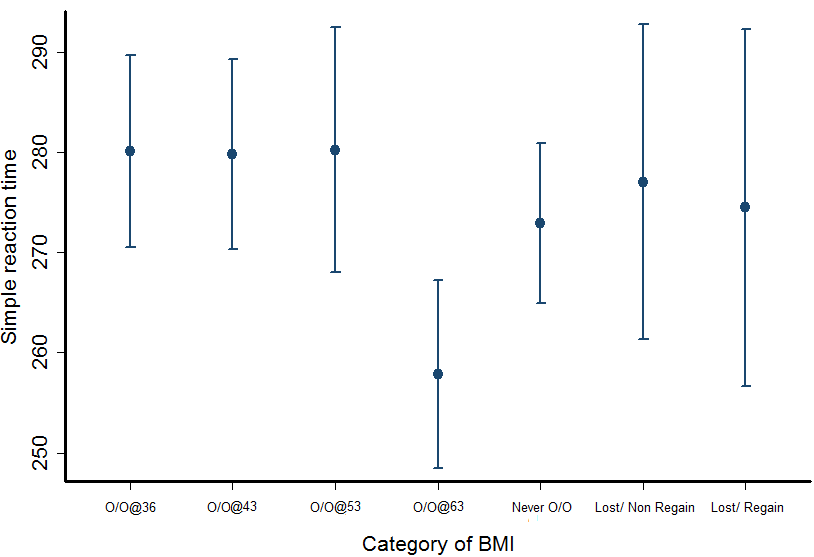

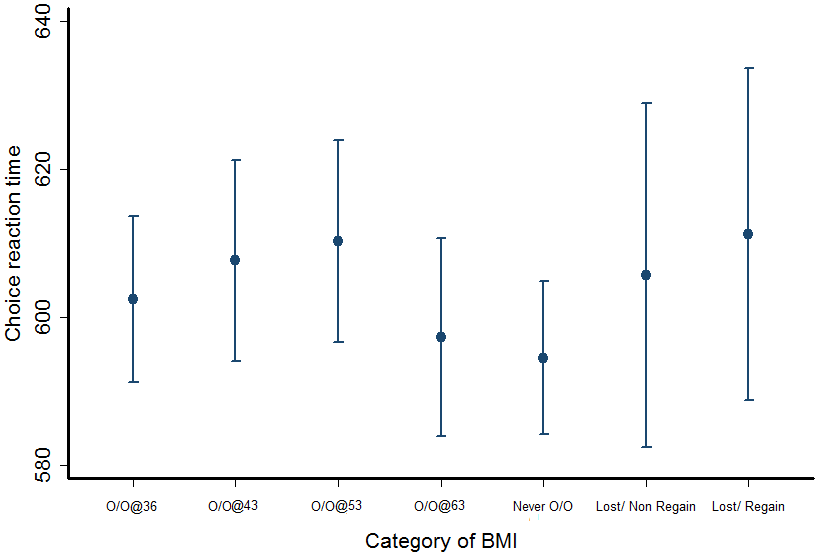


**Figure 3S. Relationship of categories of waist circumference (WC) and letter search speed test (LSST) result at age 60–64 years in the whole population.** Data points are mean reaction time in seconds and bars represent 95% CI for each group. HiWC@36, HiWC@43, HiWC@53, HiWC@60-64 = Overweight/obesity since 36,43, 53 and 60-64 years old, respectively; Never HiWC = WC always normal; Lost/Non regain = dropped and did not regained one category of WC; Lost/Regain = dropped and regained one category of WC. Results are adjusted for sex, childhood cognition, education and probability of survival at age 60-64 years. Results are adjusted for sex, childhood cognition, education and inverse probability weighting was implemented to account for drop out due to death.


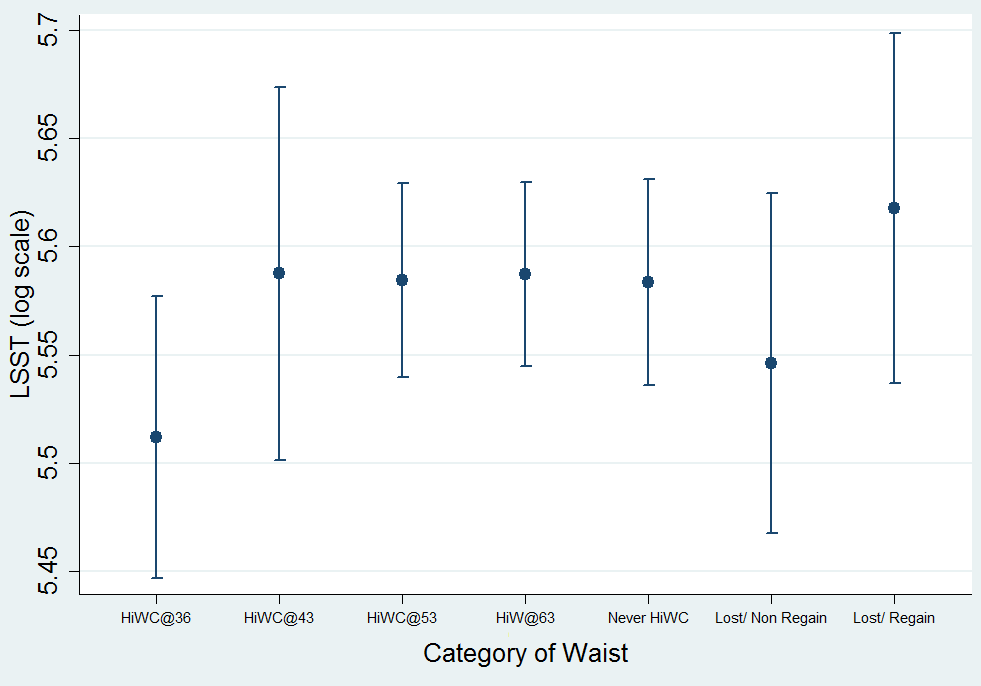


**Figure 4S. Patterns of waist circumference (WC) change and performance at the simple (A) and choice (B) reaction time tests at age 60–64 years.** Data points are mean reaction time in seconds and bars represent 95% CI for each group. HiWC@36, HiWC@43, HiWC@53, HiWC@60-64 = Overweight/obesity since 36,43, 53 and 60-64 years old, respectively; Never HiWC = WC always normal; Lost/Non regain = dropped and did not regained one category of WC; Lost/Regain = dropped and regained one category of WC. Results are adjusted for sex, childhood cognition, education and probability of survival at age 60-64 years. Results are adjusted for sex, childhood cognition, education and inverse probability weighting was implemented to account for drop out due to death.

**A) B)**


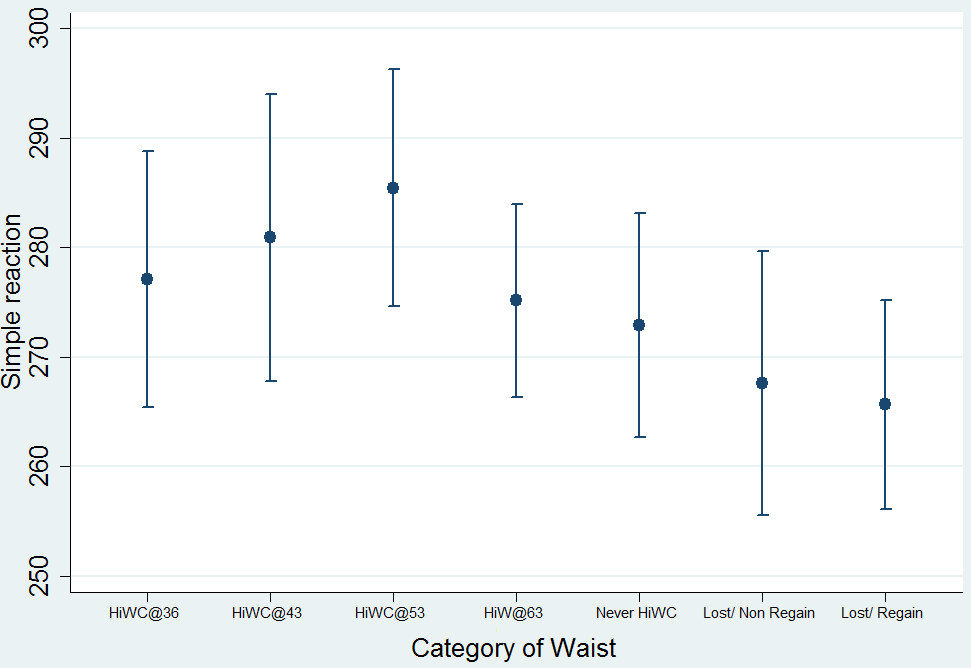

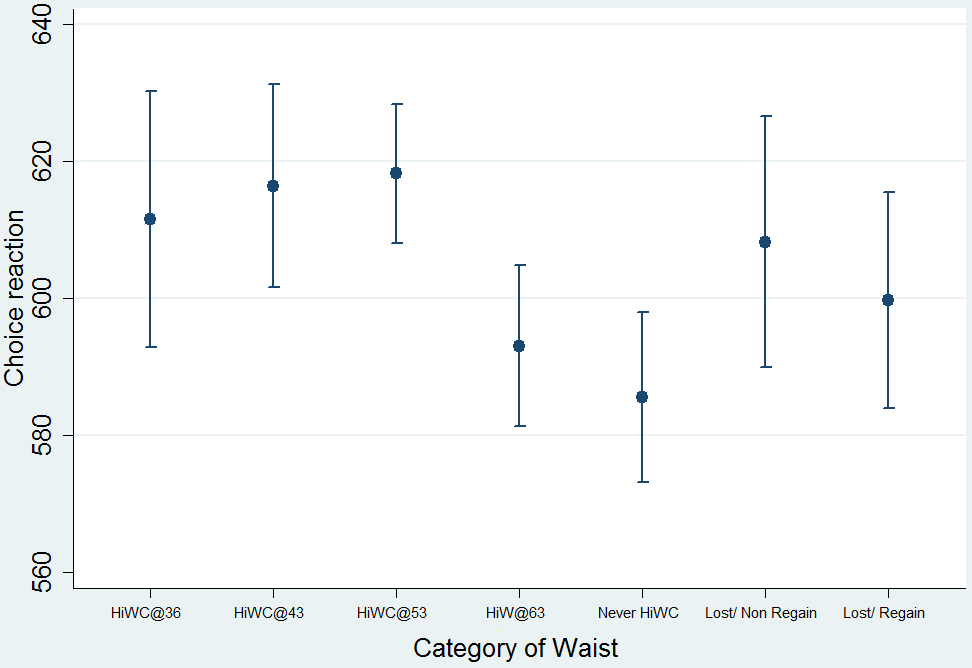


**Figure 5S.** **Scatter plots reporting the different associations of rates of changes of BMI or WC with each cognitive test.** Relationships of Rates of change of BMI at different ages with: A) verbal memory test (VMT); B) log letter search speed test (logLSST); C) simple reaction time test (S-RT); D) choice reaction time (C-RT)

**A) B)**

**
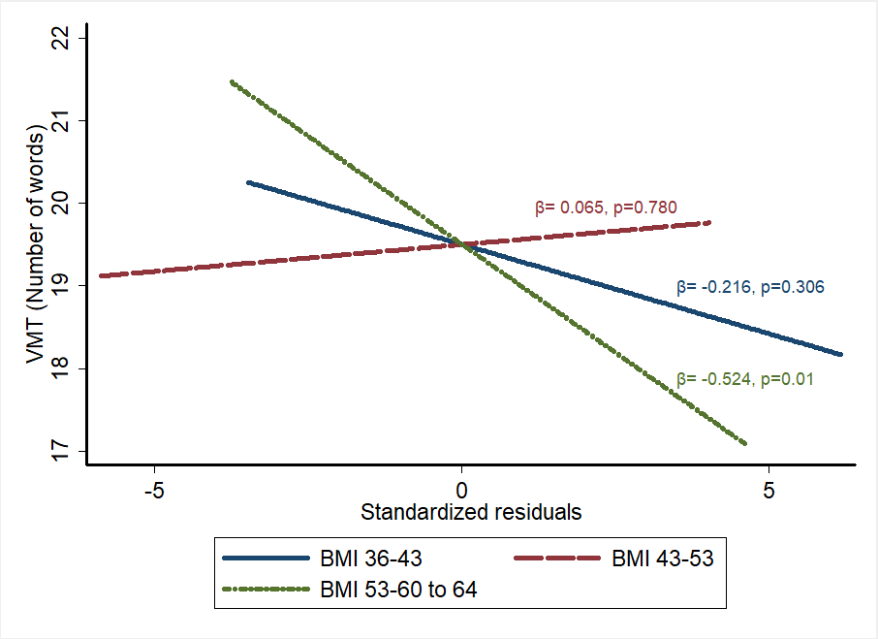

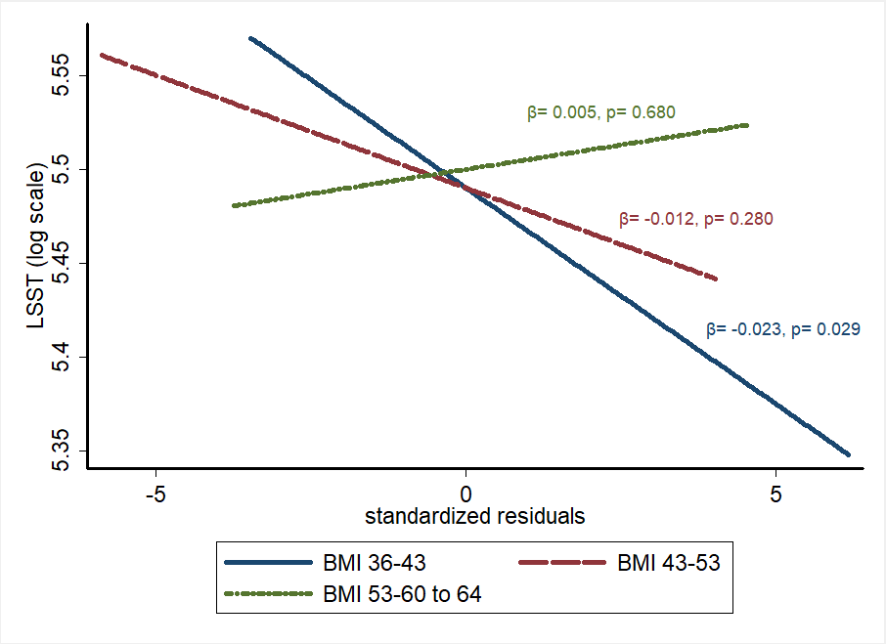
**

**C) D)**

**
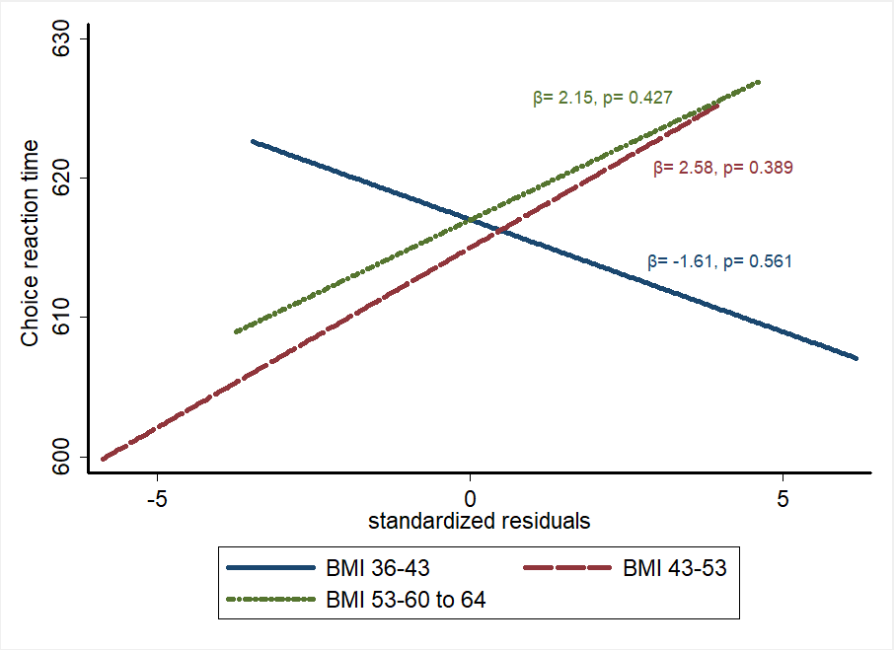

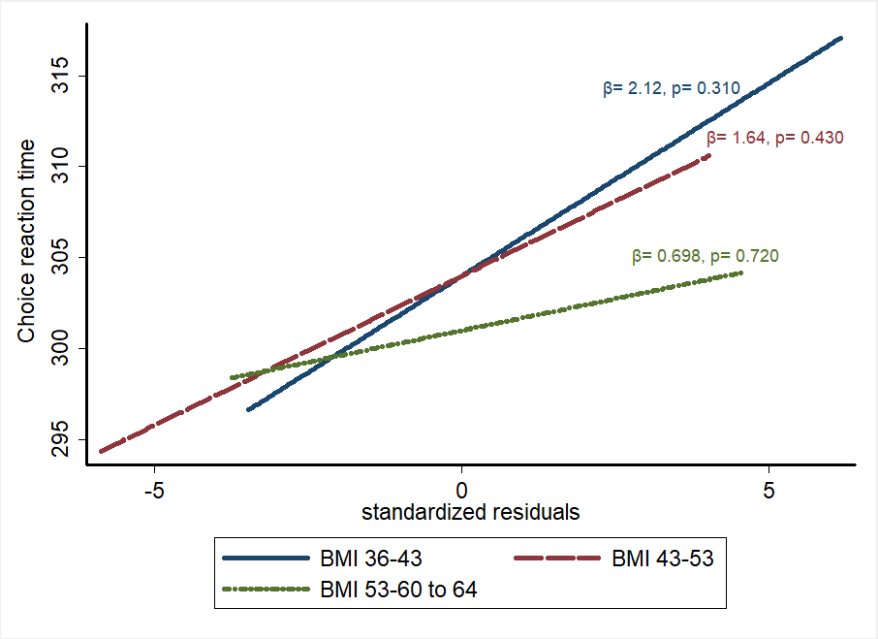
**

**Figure 6S. Association of rate of change in BMI in three time periods (36–43 years, 43–53 years and 53–60 to 64 years) and letter search speed (LSST) at age 60–64 years in females.** β coefficients represents the slope of the linear regression and indicate the difference in log LSST for 1 standard deviation increase in BMI in each interval. In each model, inverse probability weighting was implemented to account for drop out due to death.


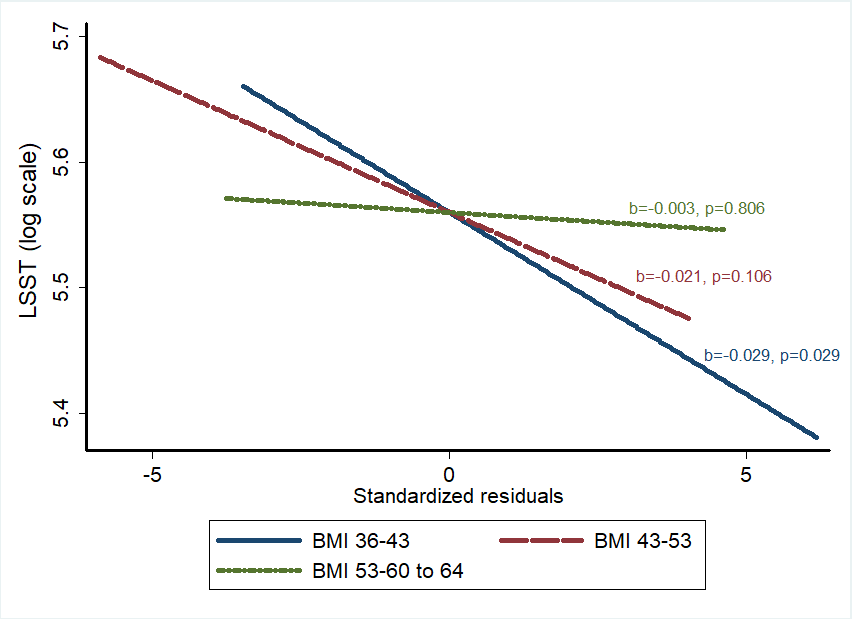


**References**

1. Stafford M, Black S, Shah I, Hardy R, Pierce M, Richards M, Wong A, Kuh D: **Using a birth cohort to study ageing: representativeness and response rates in the National Survey of Health and Development**. *European journal of ageing* 2013, **10**(2):145-157.

2. Weber T, Ammer M, Rammer M, Adji A, O'Rourke MF, Wassertheurer S, Rosenkranz S, Eber B: **Noninvasive determination of carotid-femoral pulse wave velocity depends critically on assessment of travel distance: a comparison with invasive measurement**. *Journal of hypertension* 2009, **27**(8):1624-1630.

3. Stein JH, Korcarz CE, Hurst RT, Lonn E, Kendall CB, Mohler ER, Najjar SS, Rembold CM, Post WS, American Society of Echocardiography Carotid Intima-Media Thickness Task F: **Use of carotid ultrasound to identify subclinical vascular disease and evaluate cardiovascular disease risk: a consensus statement from the American Society of Echocardiography Carotid Intima-Media Thickness Task Force. Endorsed by the Society for Vascular Medicine**. *Journal of the American Society of Echocardiography : official publication of the American Society of Echocardiography* 2008, **21**(2):93-111; quiz 189-190.

4. Tikhonoff V, Hardy R, Deanfield J, Friberg P, Kuh D, Muniz G, Pariante CM, Hotopf M, Richards M, scientific N *et al*: **Symptoms of anxiety and depression across adulthood and blood pressure in late middle age: the 1946 British birth cohort**. *Journal of hypertension* 2014, **32**(8):1590-1598; discussion 1599.

5. Skidmore PM, Hardy RJ, Kuh DJ, Langenberg C, Wadsworth ME: **Birth weight and lipids in a national birth cohort study**. *Arteriosclerosis, thrombosis, and vascular biology* 2004, **24**(3):588-594.

6. Jones R, Hardy R, Sattar N, Deanfield JE, Hughes A, Kuh D, Murray ET, Whincup PH, Thomas C, Scientific N *et al*: **Novel coronary heart disease risk factors at 60-64 years and life course socioeconomic position: the 1946 British birth cohort**. *Atherosclerosis* 2015, **238**(1):70-76.

7. Mitchell GF, van Buchem MA, Sigurdsson S, Gotal JD, Jonsdottir MK, Kjartansson O, Garcia M, Aspelund T, Harris TB, Gudnason V *et al*: **Arterial stiffness, pressure and flow pulsatility and brain structure and function: the Age, Gene/Environment Susceptibility--Reykjavik study**. *Brain : a journal of neurology* 2011, **134**(Pt 11):3398-3407.

8. Waldstein SR, Rice SC, Thayer JF, Najjar SS, Scuteri A, Zonderman AB: **Pulse pressure and pulse wave velocity are related to cognitive decline in the Baltimore Longitudinal Study of Aging**. *Hypertension* 2008, **51**(1):99-104.

9. Charakida M, Khan T, Johnson W, Finer N, Woodside J, Whincup PH, Sattar N, Kuh D, Hardy R, Deanfield J: **Lifelong patterns of BMI and cardiovascular phenotype in individuals aged 60-64 years in the 1946 British birth cohort study: an epidemiological study**. *The lancet Diabetes & endocrinology* 2014, **2**(8):648-654.

10. Department of Nutrition for Health and Development WHO: **Waist Circumference and Waist–Hip Ratio: Report of a WHO Expert Consultation** In*.*: World Health Organization 2008.

11. Wills AK, Hardy RJ, Black S, Kuh DJ: **Trajectories of overweight and body mass index in adulthood and blood pressure at age 53: the 1946 British birth cohort study**. *Journal of hypertension* 2010, **28**(4):679-686.
